# Supplementary material for: Desegregation of neuronal predictive processing
Source: Nat Commun. 2026 Mar 13;17:3919. doi: 10.1038/s41467-026-70347-w (PMC13129095; doi:10.1038/s41467-026-70347-w)
Supplement: Supplementary file 1 — Supplementary Information [file 41467_2026_70347_MOESM1_ESM.pdf]

Supplementary Information for
"Desegregation of neuronal predictive processing"

Bin Wang[1,2], Nicholas J Audette[3], David M Schneider[3], Johnatan Aljadeff[1,*]

[1] University of California San Diego [2] Columbia University [3] New York University

1 **Supplementary Figures S1-S11** **3**

2 **A normative framework for high-dimensional predictive processing** **14**

2.1 The recurrent network model 14

2.2 A Bayesian inference perspective of the network model 18

2.3 Extensions of the network model 19

2.3.1 Associations between more than two modalities 19

2.3.2 Neurons with dendritic compartments 20

2.3.3 Hierarchical network architecture 21

3 **Predictive representations in recurrent networks** **23**

3.1 Replica calculation of the firing-rate statistics 24

3.2 Single-neuron and population statistics 32

3.2.1 The high-dimensional case, $P/N \to \alpha > 0$ 32

3.2.2 The case $\alpha \to 0$ 34

3.3 Balance level distribution 35

4 **Characterizing different functional neuron types** **36**

4.1 Firing-rate correlations from two-body replica calculations 36

4.2 Explicit formulas in the Gaussian case 42

4.3 Imperfect match of paired stimuli 43

5 **The E/I network model** **45**

5.1 Derivation of the E/I connectivity in the model 45

5.2 Interpolation via nonnegative matrix factorization 48

5.3 Plasticity of inhibitory weights during learning 50

6 **Parameter values used in the figures** **51**

References **54**

1. SUPPLEMENTARY FIGURES S1-S11

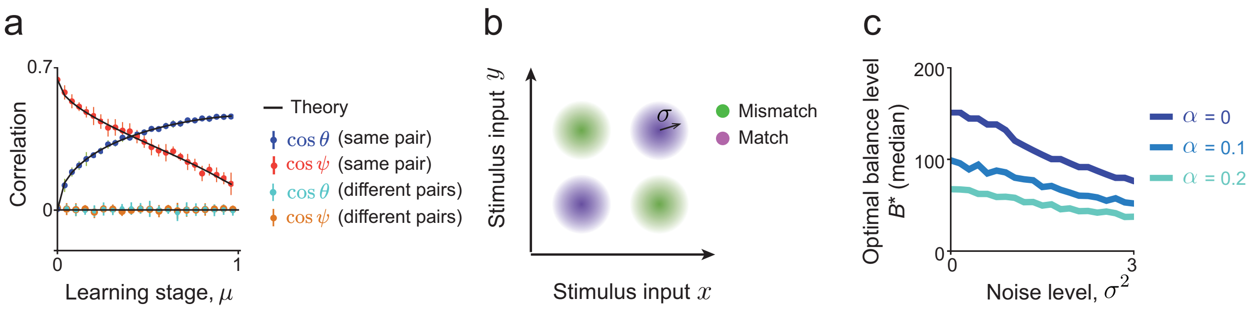

Fig. S1. The geometry of predictive representations in the model. (a) Pearson correlation coefficient between neural responses in different stimulus conditions. As in Fig. 1, the angle θ is measured between the network's responses to the two stimuli in mismatch conditions (i.e., $\boldsymbol{r}_x$ and $-\boldsymbol{r}_y$); while ψ is the angle between responses to the same stimulus in the match and mismatch conditions (i.e., $\boldsymbol{r}_{xy}$ and $\boldsymbol{r}_x$). Neural responses to stimuli from different stimulus-pairs remain uncorrelated, suggesting that the predictive signal learned by the network is stimulus-specific. Here $\alpha = 0$. Data are shown as mean values +/- SD, based on $n = 10$ repeats of network. (b) Schematic of noisy stimulus inputs. Independent isotropic Gaussian noise (with S.D. denoted by σ) is added to the inputs in the match and mismatch conditions, relative to the noiseless stimulus presentation considered in Figs. 1,2. (c) The optimal balance level decreases as stimulus presentation becomes more noisy for all values of α.

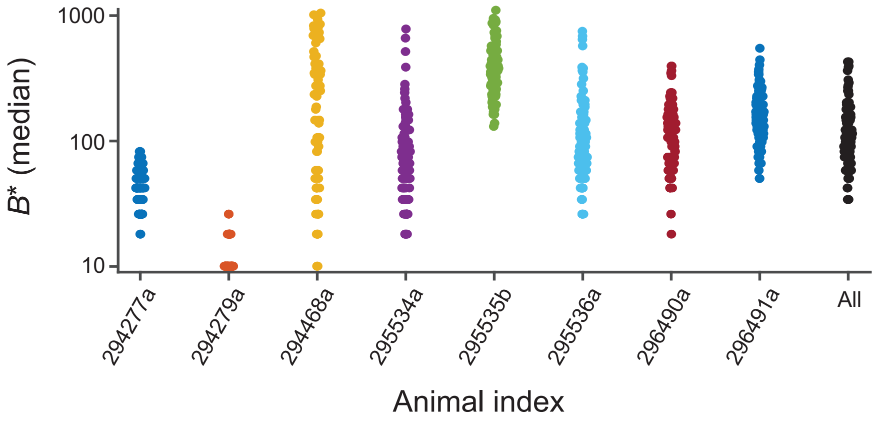

Fig. S2. Estimated balance levels from individual animals. For each animal recorded in [1] ($n = 8$), the balance level was estimated as described in the Methods, sampling the firing-rates separately from each animal. There is marked variability across animals, suggesting that effects of learning multiple stimuli in the future are best studied within animal during learning.

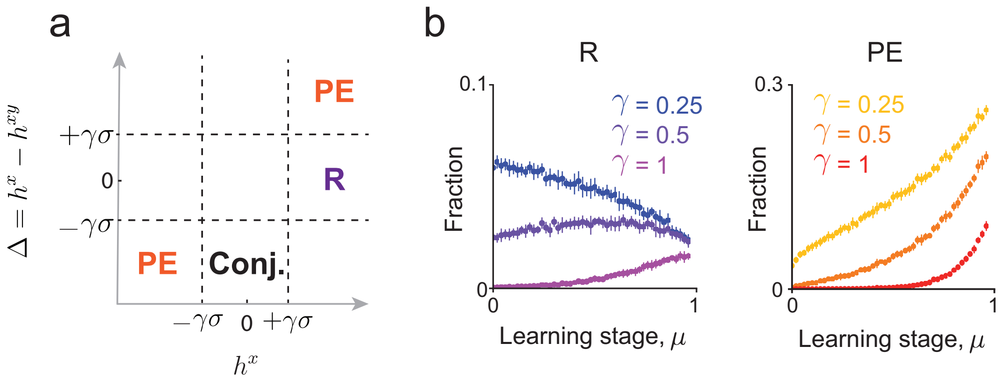

Fig. S3. Abundance of functional cell types as a function of learning stage and classification threshold. (a) Criteria for classifying different functional cell types. The classification is based on setting two thresholds ($\pm\gamma\sigma$) on the voltage response in the x-only mismatch condition (h_i^x), and its difference from the voltage response to match condition ($h_i^x - h_i^{xy}$, see Methods). The regions corresponding to prediction-error (PE) and representation (R) neurons for stimulus x are shown in the plot. Here we do not distinguish positive or negative PE neurons. Similar criteria are applied when replacing x with y. Also shown is the region corresponding to the conjunctive (Conj.) neurons, which have a small response in x-only mismatch condition but a large response in the match condition. (b) Fraction of R and PE neurons for different threshold values, as a function of the learning stage μ. The fraction of PE neurons increases during learning independently of the threshold. Here $\alpha = 0$. Circles: mean; Error-bars: 1 S.D. computed over $n = 10$ instances of the network.

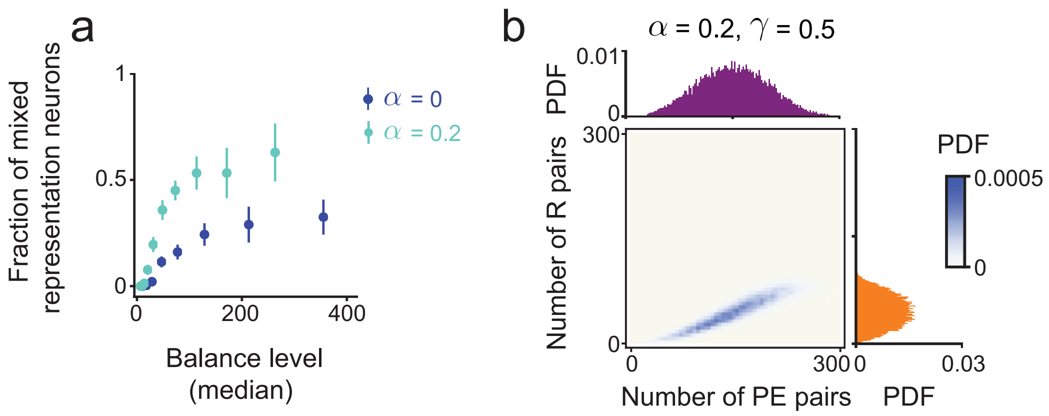

Fig. S4. Fraction of mixed-representation neurons as a function of balance level and stimulus dimensionality α. (a) Here we vary the gain parameter b to generate a range of balance levels (median). As the stimulus dimensionality α increases, the fraction of mixed representation neurons for a fixed balance level also increases. Circles: mean; Error-bars: 1 S.D. computed over $n = 10$ instances of the network. (b) Each neuron in the network is a representation neuron for a certain number of stimulus-pairs ('Number of R pairs') and a prediction-error neuron for other stimulus-pairs ('Number of PE pairs'). Plotted is the joint distribution of these two numbers for neurons in a network when it is trained to associate $P = 400$ stimulus-pairs. The corresponding marginal distributions are also shown. The joint distribution has a positive correlation. This indicates that when all P stimulus-pairs are considered, more neurons have a mixed representation than would be expected if the representation of stimulus and prediction-error was independent across pairs.

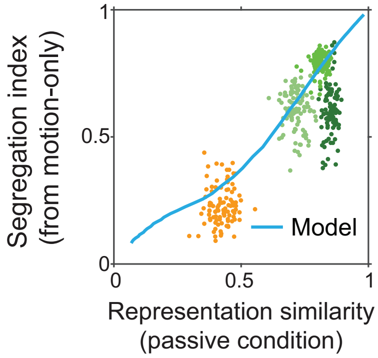

Fig. S5. Segregation index as a function of representation similarity for different pairs of expected and probe sounds. Plotted are the segregation indices as a function of the representation similarity for different probe types (similar to Fig. 4f for $n = 5$ animals). Here the segregation indices are computed based on the differences Δ between the motion-only mismatch (passive: movement-only) and match (active: lever press + sound) neural responses. Colored points correspond to subsamples of the data. The results exhibit a similar trend as in Fig. 4f. The model curve shown in this plot is computed using a different sparsity level (by varying the firing threshold θ) compared to the values used in Fig. 4f. Under our main modeling assumptions: connectivity that is symmetric and puts the stimuli x and y on 'equal footing' during learning, synaptic weights with Gaussian statistics, and ReLU nonlinearity, we were not able to find a single value of θ to fit the data with two definitions of mismatch responses. Future work with more realistic network connectivity may give a choice of parameters that is consistent across both ways of comparing neural responses in expected and unexpected stimulus conditions.

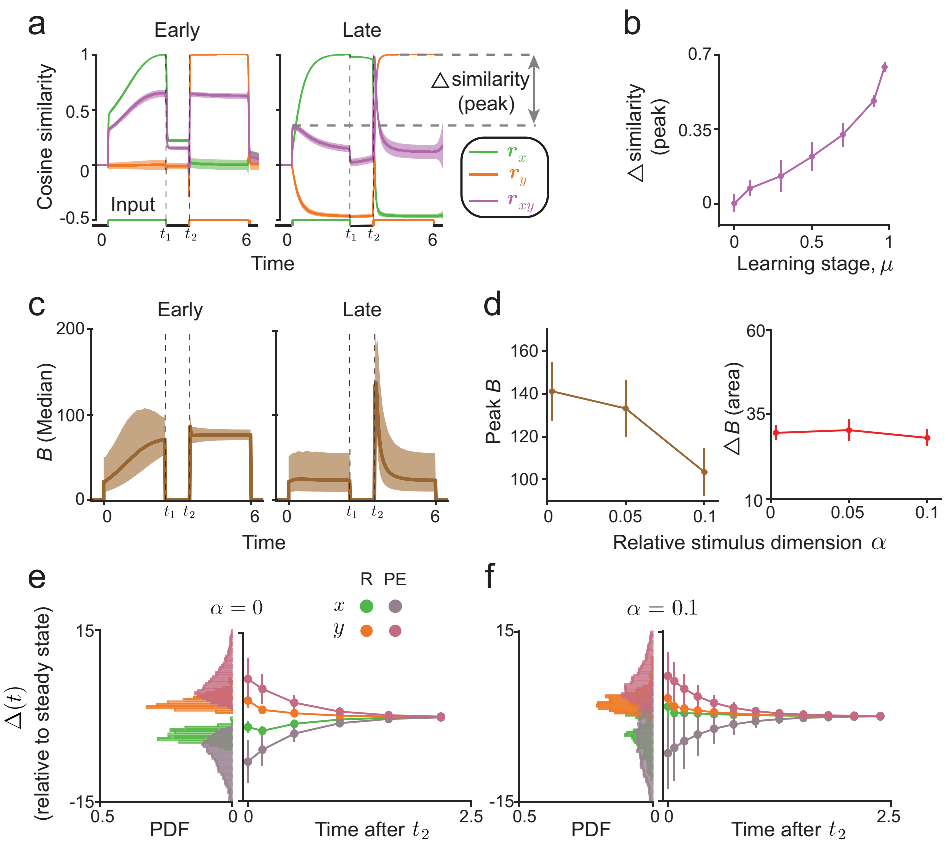

Fig. S6. Predictive representations of transient and constant high-dimensional stimuli are similar. (a) Cosine similarity between the time-dependent firing rate vector to step-like input and the corresponding steady state response (in the x-only, y-only and match conditions), shown for early and late stages of learning. ($\mu = 0,\ 0.9$). After learning, the neural response is transiently similar to steady state match responses at the onset of y stimulus, suggesting that the network predicts the presence of y based on the presentation of x even after it is removed. (b) Learning increases the 'excess peak similarity' to the the steady-state match response (r_{xy}) gained when y is presented relative to transient presentation of x. (c) The median of the time-dependent balance level B. After learning, tight balance emerges transiently at the onset of the second (y) stimulus, a signature of the transient predictive computation. (d) The peak of the median balance level decreases as the stimulus dimension increases (left). The area under the median B curve is insensitive to stimulus dimension (right). (e, f) Time-dependent voltage difference $\Delta(t)$ across different functional cell-types after onset of the second (y) stimulus. Functional cell-types are defined based on steady state responses. Here Δ for each neuron is calculated as the difference between the voltage at time t and the steady state voltage level in the y-only condition. The network exhibits cell-type specific response distributions immediately after the onset of the second (i.e., the predicted) stimulus (left). Differences between the average responses of different functional cell-types last for ~ 1 membrane time constant (right). Lines: mean values; Error-bars/shaded area: 1 S.D., computed over $n = 50$ instances of the network.

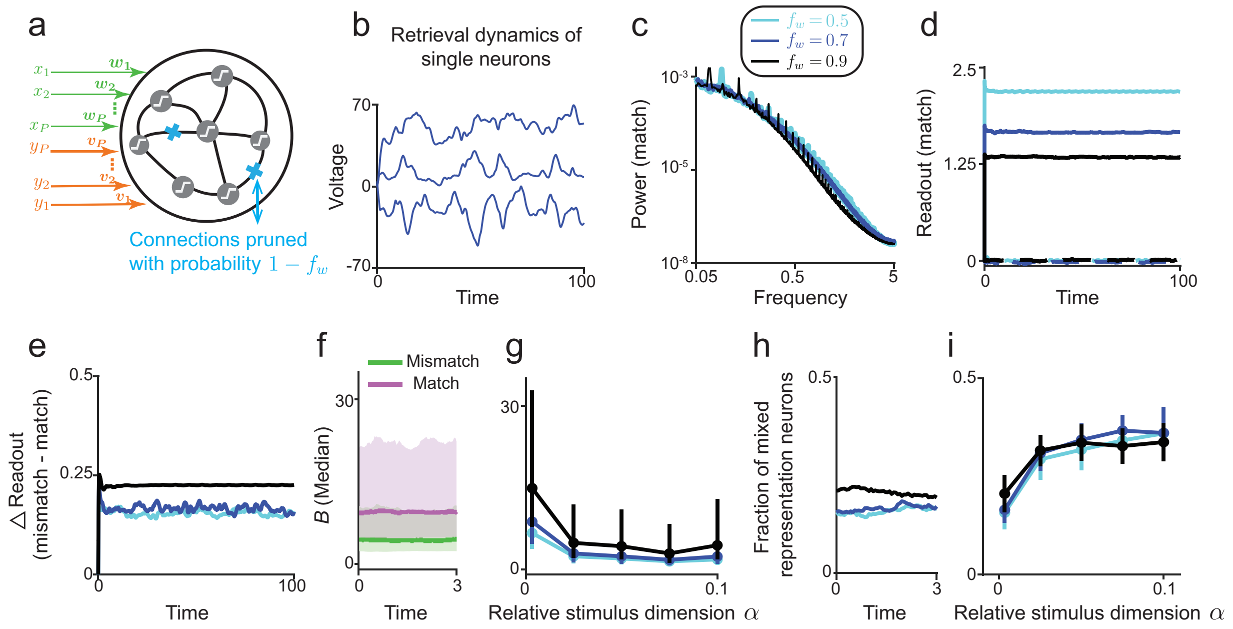

Fig. S7. Predictive processing in the model is robust when connectivity is sparse and asymmetric. (a) Schematic of the network model where synaptic connections of the fully-connected model are pruned randomly with probability $1 - f_w$. An upper bound is imposed to the nonlinear activation function to prevent runaway firing rates. (b, c) Neural activity exhibits sustained fluctuations with a broad power spectrum due to the asymmetric connectivity. (d) Linear readouts of internal predictions ($\hat{x}$, $\hat{y}$) remain stable over time despite the ongoing fluctuations. Colors correspond to different degrees of sparsity. Dashed lines show readouts of predictions of non-paired stimuli, indicating that predictions are stimulus-specific. (e) The difference between the readout of stimulus predictions in the mismatch and match conditions is also sustained despite ongoing fluctuations. (f) The median balance level exhibits fluctuations, yet the distributions of B in the match and mismatch conditions are non-overlapping. Here, $f_w = 0.7$. Lines: median; Shaded area: IQR. (g) As the stimulus dimension increases, the overall balance level decreases. Circles and error-bars: mean $\pm$ 1 S.D., computed over $n = 10$ instances of the network. (h) The fraction of mixed-representation neurons fluctuates but remains approximately constant over time. The threshold for defining functional cell-types is based on the standard deviation of voltage levels in the mismatch condition at $\alpha = 0$ and $\mu = 0.97$. (i) The fraction of mixed representation neurons in the network increases as the stimulus dimension increases. Circles and error-bars: mean $\pm$ 1 S.D., computed over $n = 10$ instances of the network.

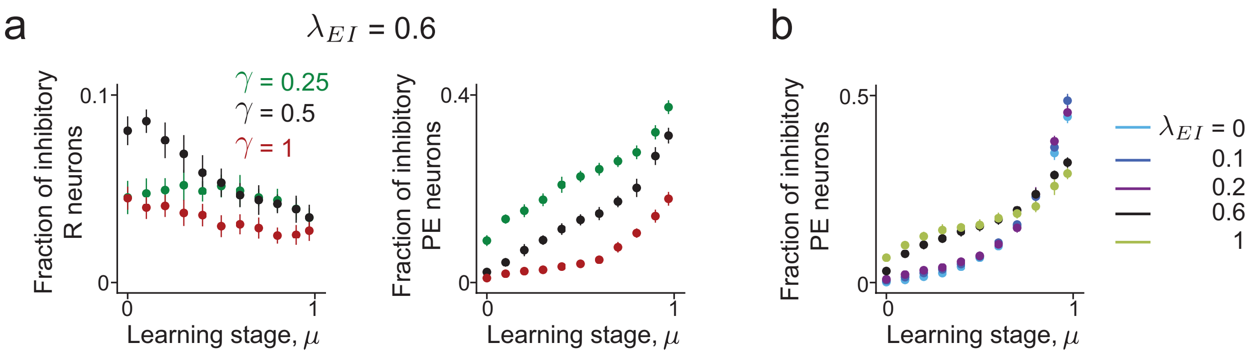

Fig. S8. Abundance of functional cell types among inhibitory neurons. (a) Fraction of inhibitory representation (R) and prediction-error (PE) neurons at different learning stages (different values of μ) when using different voltage thresholds ($\pm\gamma\sigma$). For the connectivity parameter that best matches our data ($\lambda_{EI} = 0.6$), the effect of learning is consistent across different thresholds. (b) Fraction of inhibitory prediction-error neurons at different learning stages for different values of λ. Unlike other network properties that do depend on the architecture of inhibitory connectivity (shown in Fig. 5), this quantity depends weakly on the parameter λ_{EI}. In this plot we set $\alpha = 0$. Circles and error-bars: mean $\pm$ 1 S.D., computed over $n = 20$ instances of the network.

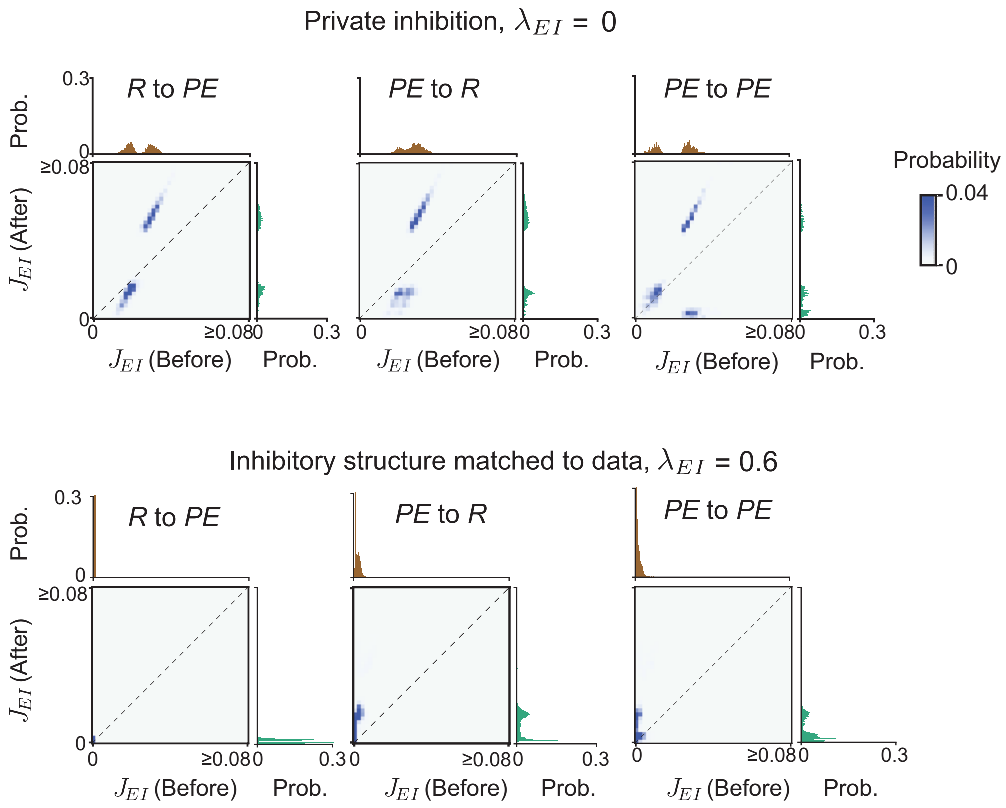

Fig. S9. Changes to inhibitory to excitatory connections during learning do not depend strongly on the functional cell type of the target. Synaptic weight distribution of I-to-E connections before and after learning, when $\lambda_{EI} = 0$ (top) and $\lambda_{EI} = 0.6$ (bottom), for pairs of E and I neurons belonging to different functional classes: (R to PE, left; PE to R, middle; PE to PE, right). These fine-scale distributions show similar trends as in Fig. 6f,g.

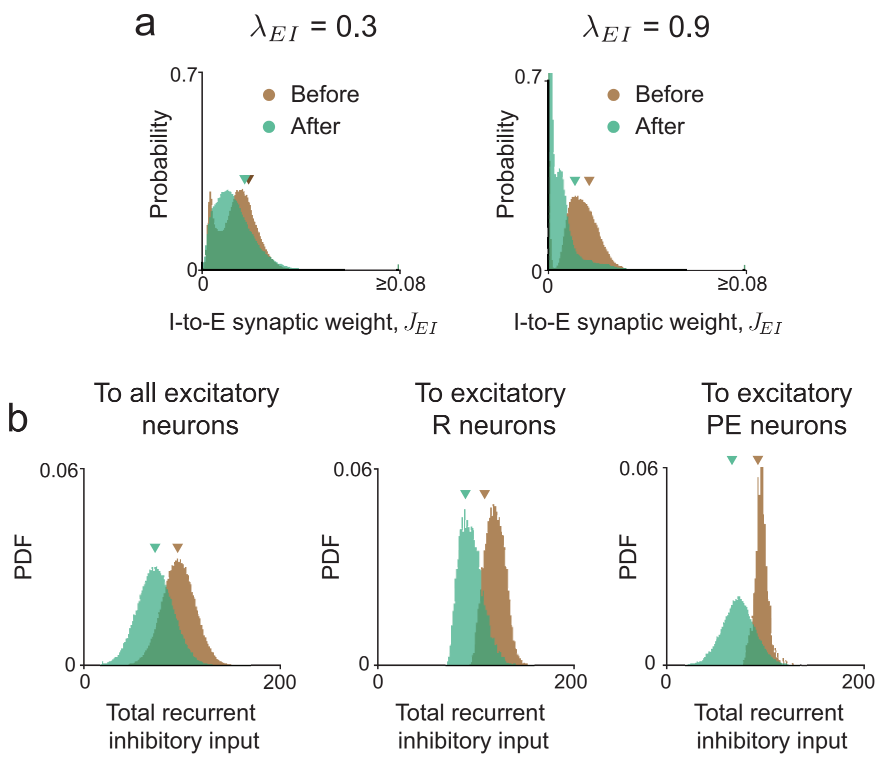

Fig. S10. Learning predictive representations does not rely on overall potentiation of inhibitory connections, across different network architectures. (a) Synaptic weight distribution of all I-to-E connections before and after learning for values of λ_{EI} not shown in Fig. 6. There is no overall increase in the strength of inhibitory synapses after learning, suggesting that across different network architectures, predictive computations that lead to suppressed responses to expected stimuli are distributed. (b) Distribution of the total recurrent inhibitory input received by different populations of excitatory neurons, in the match condition. The overall inhibition received by excitatory neurons in the network decreases after learning.

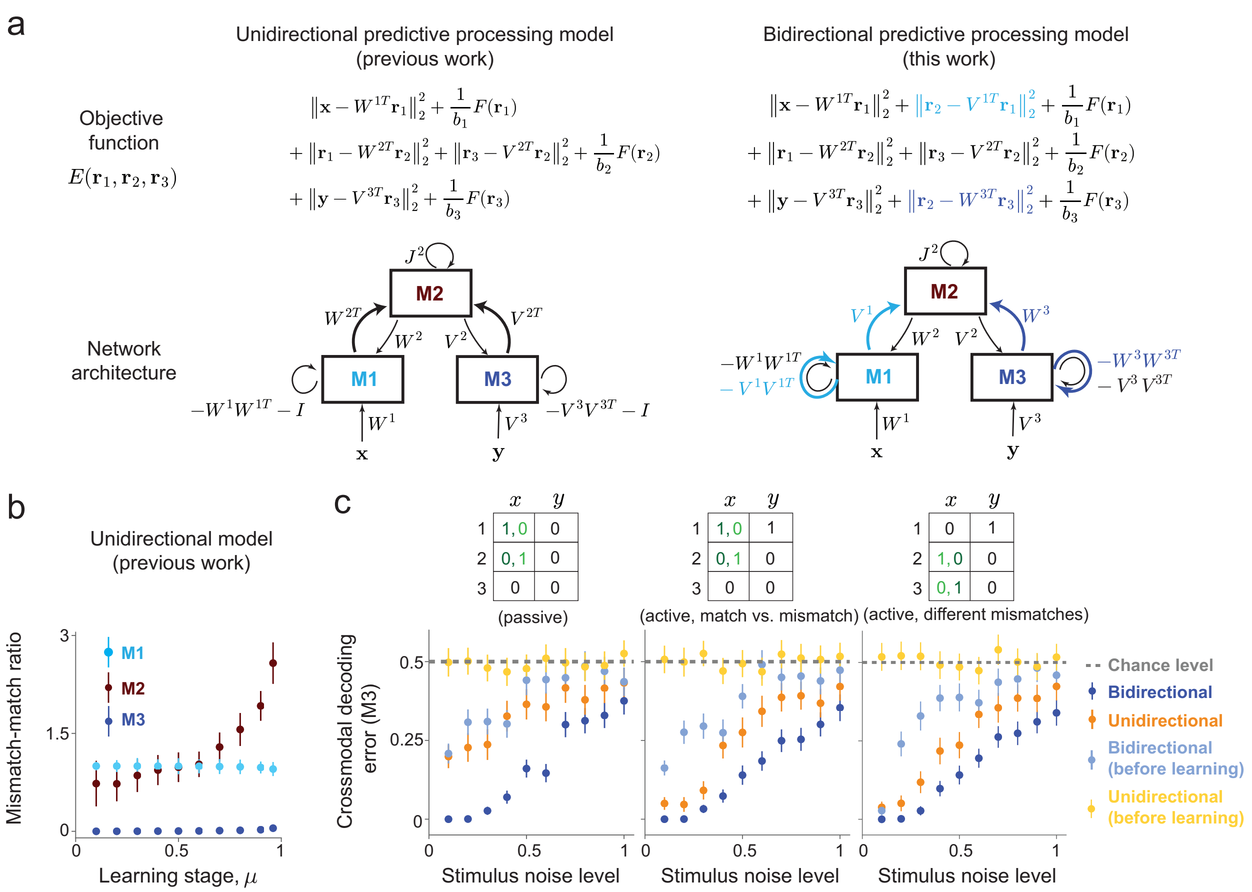

Fig. S11. Comparison between predictive representations in unidirectional and bidirectional predictive processing models. (a) The objective functions of the two models differ. In the bidirectional model, M1 and M3 generate predictions for activity in M2, and the corresponding prediction-errors are minimized (terms highlighted in light, dark blue). These differences in the objective functions imply distinct network architectures. Specifically, the recurrent connections within M1 and M3, as well as the feedforward connections (M1→M2, M3→M2), differ. In the cartoons showing network architectures, we omitted the constant term $\frac{b_l}{N}$ in the connectivity for simplicity. (b) Throughout training, M3 in the unidirectional model shows very weak mismatch responses in the x-only mismatch condition, relative to the matched condition. This contrasts with the bidirectional model (Fig. 7c), in which robust cross-modal responses in M3 emerge during training. (c) Decoding errors of stimulus-x information based on activity of M3 neurons (i.e., the cross-modal decoding error). We compared the unidirectional and bidirectional models under different stimulus conditions involving 3 stimulus dimensions (see table above each panel). Cross-modal decoding errors are consistently smaller in the bidirectional model, indicating stronger cross-modal communication across modules. Left: decoding between conditions where only x_1 or only x_2 is presented. Middle: same as left, but with stimulus y_1 also presented. Right: decoding between mismatches along different x dimensions (x_2, x_3 paired with y_1). Circles and error-bars: mean $\pm$ 1 S.D., computed over $n = 50$ instances of the network.

2. A NORMATIVE FRAMEWORK FOR HIGH-DIMENSIONAL PREDICTIVE PROCESSING

2.1. The recurrent network model

We consider a network of N recurrently connected neurons, where the firing-rates of the neurons are denoted by the vector $\boldsymbol{r}(t) = (\, r_1(t), \ldots, r_N(t)\,)$. The firing-rate of each neuron is related to its voltage level h_i via a nonlinear activation function, $r_i(t) = \phi(h_i(t))$. We denote the learned paired inputs to the network as $\boldsymbol{x}(t) = (\, x^1(t), \ldots, x^P(t)\,)$ and $\boldsymbol{y}(t) = (\, y^1(t), \ldots, y^{P'}(t)\,)$. Notice that the dimensions of the paired inputs are not necessarily the same in this section.

In the predictive coding framework, the network continuously generates an internal prediction of the inputs. We assume that internal predictions (denoted $\hat{x}^k(t)$, $\hat{y}^k(t)$) are linear read-outs from the network activity, i.e.,

$$\begin{aligned} \hat{x}^k(t) &= \frac{1}{N}\boldsymbol{w}^k \cdot \boldsymbol{r}(t), \quad k = 1, \ldots, P, \\ \hat{y}^{k'}(t) &= \frac{1}{N}\boldsymbol{v}^{k'} \cdot \boldsymbol{r}(t), \quad k' = 1, \ldots, P'. \end{aligned} \tag{S1}$$

Here $\boldsymbol{w}^k, \boldsymbol{v}^{k'}$ are the N-dimensional readout weight vectors.

Our aim is to derive a network model where the prediction-errors are minimized subject to some regularization term on encoding efficiency. Mathematically, we define the following objective function,

$$E(t) = \sum_{k=1}^{P} \left(x^k(t) - \hat{x}^k(t)\right)^2 + \sum_{k=1}^{P'} \left(y^k(t) - \hat{y}^k(t)\right)^2 + \frac{2}{bN}\sum_{i=1}^{N} F(r_i(t)). \tag{S2}$$

The first two terms of $E(t)$ correspond to the prediction-errors. The regularization term, and the function $F(z)$ in particular, depend on the nonlinear activation function ϕ. We consider those nonlinear activation functions where the firing-rate is $\phi_+(h - \theta)$ above a threshold θ, and 0 below the threshold. Mathematically,

$$\phi(h) = \begin{cases} \phi_+(h-\theta) & \text{if } h \geq \theta, \\ 0 & \text{if } h < \theta. \end{cases} \tag{S3}$$

Here ϕ_+ is a monotonically increasing smooth function which vanishes at 0, such that ϕ is continuous. This class of functions includes a number of activation functions used in previous work, e.g., rectified linear activation (ReLU, $\phi_+(h) = h$) and rectified nonlinear units, $\phi_+(h) = h^p$ $(p > 0)$, that coincide with ReLU for $p = 1$.

For this choice of ϕ, we show below that the recurrent network dynamics

$$\tau \frac{\mathrm{d}h_i(t)}{\mathrm{d}t} = -h_i(t) + \sum_{j=1}^{N} J_{ij}\phi(h_j(t)) + \sum_{k=1}^{P} b\, w_i^k x^k + \sum_{k'=1}^{P'} b\, v_i^{k'} y^{k'}, \tag{S4}$$

with the connectivity matrix and choice of regularization,

$$\begin{aligned} J_{ij} &= -\frac{b}{N}\left(\sum_{k=1}^{P} w_i^k w_j^k + \sum_{k'=1}^{P'} v_i^{k'} v_j^{k'}\right), \\ F(r) &= \int_0^r \phi_+^{-1}(z)dz + \theta r = \frac{p}{p+1} r^{1+\frac{1}{p}} + \theta r, \end{aligned} \tag{S5}$$

minimizes the objective [Eq. (S2)]. Note that adding a nonzero firing threshold ($\theta > 0$) in the regularization function enforces sparse neural responses, penalizing large firing-rates. For the ReLU nonlinearity ($p = 1$), we have $F(r) = r^2/2 + \theta r = (r+\theta)^2/2 - \theta^2/2$.

We assume that the timescale of changes to the inputs is much slower than the timescale of changes to neuronal activity, such that we can ignore potential time-dependencies of $\boldsymbol{x}$ and $\boldsymbol{y}$. Under this assumption, the objective [Eq. (S2)] can be written as a function of the neural activity and readout weights,

$$E(\boldsymbol{r}; \{\boldsymbol{w}^k, \boldsymbol{v}^k\}) = \sum_{k=1}^{P}\left[\left(x^k - \frac{1}{N}\boldsymbol{w}^k\cdot\boldsymbol{r}\right)^2 + \left(y^k - \frac{1}{N}\boldsymbol{v}^k\cdot\boldsymbol{r}\right)^2\right] + \frac{2}{bN}\sum_{i=1}^{N} F(r_i). \tag{S6}$$

The neural activity $\boldsymbol{r}(t)$ governed by the dynamical equations [Eq. (S4)] with the connectivity matrix [Eq. (S5)] minimizes the objective function [Eq. (S2)]. This can be shown by directly evaluating the time derivative of $E(t)$:

$$\begin{aligned} \frac{\mathrm{d}E(t)}{\mathrm{d}t} &= \sum_{i=1}^{N} \frac{\partial E}{\partial r_i}\frac{\partial r_i}{\partial h_i}\frac{\mathrm{d}h_i}{\mathrm{d}t} \\ &= -\sum_{i=1}^{N}\left[2\sum_{k=1}^{P}(x^k - \hat{x}^k)\frac{w_i^k}{N} + 2\sum_{k'=1}^{P'}(y^{k'} - \hat{y}^{k'})\frac{v_i^{k'}}{N} - \frac{2}{bN}\phi_+^{-1}(r_i) - \frac{2\theta}{bN}\right]\phi'(h_i)\frac{\mathrm{d}h_i}{\mathrm{d}t} \\ &= -\frac{2}{bN}\sum_{i=1}^{N}\phi'(h_i)\frac{\mathrm{d}h_i}{\mathrm{d}t} \\ &\quad \times \left[\sum_{k=1}^{P} b w_i^k x^k + \sum_{k=1}^{P'} b v_i^{k'} y^{k'} - \frac{b}{N}\sum_{j=1}^{N}\left(\sum_{k=1}^{P} w_i^k w_j^k + \sum_{k'=1}^{P'} v_i^{k'} v_j^{k'}\right)\phi(h_j) - \phi_+^{-1}(r_i) - \theta\right] \\ \circledast &= -\frac{2}{bN}\sum_{i=1}^{N}\phi'(h_i)\frac{\mathrm{d}h_i}{\mathrm{d}t} \end{aligned}$$

$$
\times \left[\sum_{k=1}^{P} b w_i^k x^k + \sum_{k=1}^{P'} b v_i^{k'} y^{k'} - \frac{b}{N} \sum_{j=1}^{N} \left(\sum_{k=1}^{P} w_i^k w_j^k + \sum_{k'=1}^{P'} v_i^{k'} v_j^{k'} \right) \phi(h_j) - h_i \right]
$$

$$
= -\frac{2}{bN\tau} \sum_{i=1}^{N} \left(\frac{\mathrm{d}h_i}{\mathrm{d}t} \right)^2 \phi'(h_i) \tag{S7}
$$

In the line indicated by $\circledast$ we used the identity $\phi_+^{-1}(r)\phi'(h) = (h - \theta)\phi'(h)$. Each term in the sum that appears in the last line of Eq. (S7) is positive, so the time derivative of $E(t)$ is negative. The existence of Lyapunov function for Eq. (S4) indicates that the network will reach a (stable) fixed point which satisfies for each neuron i,

$$
h_i^\star = \sum_{j=1}^{N} J_{ij} \phi(h_j^\star) + \sum_{k=1}^{P} b\, w_i^k x^k + \sum_{k'=1}^{P'} b\, v_i^{k'} y^{k'}. \tag{S8}
$$

Moreover, since $E(\boldsymbol{r})$ is a strictly convex function of the firing-rate vector $\boldsymbol{r}$, the optimal fixed-point solution $\boldsymbol{r}^\star$ is unique. From Eq. (S8), $\boldsymbol{h}^\star$ is also unique. Furthermore, that fixed point is a global minimum of E, which can be shown by evaluating the first-order derivatives of Eq. (S2) at the fixed point. Taken together, our results show that the network is guaranteed to reach a stable fixed-point for any input combination (indicated by x^k and y^k), which is the minimum of Eq. (S2).

In the following sections, we will assume that there are P distinct pairs of stimuli indexed by k, (x^k, y^k). The corresponding feedforward weight vectors $\boldsymbol{w}^k, \boldsymbol{v}^k$ are assumed to be random, with mean 0. Associative training induces correlations between each component of the feedforward weights, via, for example, Hebbian-type plasticity. More precisely, for $i, j = 1, \ldots, N$ and $k, k' = 1, \ldots, P$,

$$
\langle w_i^k \rangle = \langle v_i^k \rangle = 0, \qquad \langle w_i^k w_j^{k'} \rangle = \langle v_i^k v_j^{k'} \rangle = \delta_{kk'} \delta_{ij}, \qquad \langle w_i^k v_j^{k'} \rangle = \delta_{kk'} \delta_{ij} \mu^k. \tag{S9}
$$

Here $\langle \cdots \rangle$ denotes the expectation over the probability distribution of synaptic weights. To study how neural representations change during learning we vary μ^k systematically. Note that we have rescaled μ^k by N^{-1} relative to the notation used in the main text.

Our choice of synaptic weight statistics [Eq. (S9)] arises from an optimization procedure that minimizes the objective function [Eq. (S2)]. Indeed, performing gradient descent on E within a short time window Δt induces the following weight changes,

$$
\begin{aligned}
\Delta w_i^k &= -\eta \tfrac{\partial E(\boldsymbol{r}; \{\boldsymbol{w}^k, \boldsymbol{v}^k\})}{\partial w_i^k} \Delta t = \tfrac{\eta}{N} \left(x^k - \tfrac{1}{N} \boldsymbol{w}^k \cdot \boldsymbol{r} \right) \phi(h_i) \Delta t \equiv \frac{\eta}{N} \delta x^k r_i \Delta t, \\
\Delta v_i^k &= -\eta \tfrac{\partial E(\boldsymbol{r}; \{\boldsymbol{w}^k, \boldsymbol{v}^k\})}{\partial v_i^k} \Delta t = \tfrac{\eta}{N} \left(y^k - \tfrac{1}{N} \boldsymbol{v}^k \cdot \boldsymbol{r} \right) \phi(h_i) \Delta t \equiv \frac{\eta}{N} \delta y^k r_i \Delta t.
\end{aligned} \tag{S10}
$$

We assume that the learning rate is small $\eta \ll 1$, such that the neural dynamics [Eq. (S4)]

remain at the steady state $\boldsymbol{r}^{\star}$. We will show below (SI §3.1) that during associative learning ($x^k = y^k = 1$), the variables representing prediction errors are non-negative ($\delta x^k, \delta y^k \geq 0$), which implies that the weights could grow unbounded during learning.

To prevent this potential blow-up, we introduce a normalization mechanism that regularizes the weights. After each 'learning-step' [Eq. S10], the weights change according to a 'homeostatic-step',

$$w_i^k(t) \rightarrow \frac{w_i^k(t) - m_w^k}{\sigma_w^k}, \qquad m_w^k = \frac{1}{N}\sum_{i=1}^{N} w_i^k(t), \qquad (\sigma_w^k)^2 = \frac{1}{N}\sum_{i=1}^{N}(w_i^k(t) - m_w^k)^2. \tag{S11}$$

Here m_w^k and σ_w^k are the means and the standard deviations of the weight vector $\boldsymbol{w}$ computed over the N neurons. Similar updates are applied to the weights $\boldsymbol{v}$. We show that under these update rules, $\mu^k(t)$, the correlation between $\boldsymbol{w}^k$ and $\boldsymbol{v}^k$ at time t during the learning process, increases monotonically.

We first note that the homeostatic step [Eq. (S11)] ensures that weight vectors have zero mean and unit variance. Upon presentation of the stimulus-pair k, the steady-state input to neuron i is independent of inputs to other neurons. Additionally, in the $N \rightarrow \infty$ limit, $\delta x^{k'}$ and $\delta y^{k'}$ are nonzero only if $k' = k$. These properties are shown explicitly using a replica calculation below (SI §3.1). It is therefore sufficient to verify that applying the learning-step [Eq. (S10)] does not lead to a decrease in the correlation. This can be done by a direct calculation of the correlation in Eq. (S11). Notice that in the $N \rightarrow \infty$ limit,

$$\begin{aligned}
m_w^k &\rightarrow \left\langle \Delta w_i^k \right\rangle, \\
(\sigma_w^k)^{-1} &\rightarrow \left\langle (w_i^k(t) + \Delta w_i^k - m_w^k)^2 \right\rangle^{-1/2} \\
&\rightarrow (1 + 2\left\langle w_i^k \Delta w_i^k \right\rangle + O(\Delta t^2))^{-1/2} \\
&= 1 - \left\langle w_i^k \Delta w_i^k \right\rangle + O(\Delta t^2) \\
&= 1 - \eta \hat{x}^k \delta x^k \Delta t + O(\Delta t^2).
\end{aligned} \tag{S12}$$

Therefore the weight w_i^k after the learning and homeostatic steps is,

$$\begin{aligned}
w_i^k(t + \Delta t) &= \frac{w_i^k(t) + \Delta w_i^k - m_w^k}{\sigma_w^k} \\
&= (w_i^k(t) + \Delta w_i^k - \left\langle \Delta w_i^k \right\rangle)(1 - \eta \hat{x}^k \delta x^k \Delta t) + O(\Delta t^2) \\
&= w_i^k(t) + \Delta w_i^k - \left\langle \Delta w_i^k \right\rangle - \eta w_i^k(t) \hat{x}^k \delta x^k \Delta t + O(\Delta t^2).
\end{aligned} \tag{S13}$$

Using this approximation and a similar expression for $v_i^k(t + \Delta t)$, the correlation is now,

$$\mu^k(t + \Delta t) = \left\langle w_i^k(t + \Delta t) v_i^k(t + \Delta t) \right\rangle$$

$$= \mu^k(t) + \left\langle w_i^k \Delta v_i^k(t) + v_i^k \Delta w_i^k(t) \right\rangle - \eta \mu^k(t)(\hat{x}^k \delta x^k + \hat{y}^k \delta y^k)\Delta t + O(\Delta t^2)$$
$$= \mu^k(t) + \eta[\hat{y}^k(\delta x^k - \delta y^k \mu^k(t)) + \hat{x}^k(\delta y^k - \delta x^k \mu^k(t))]\Delta t + O(\Delta t^2). \qquad \text{(S14)}$$

In the match condition ($x^k = y^k = 1$) we have from symmetry that $\hat{x}^k = \hat{y}^k$ and $\delta x^k = \delta y^k$. We will show in SI §3.1 using a replica calculation that $\hat{x}^k, \delta x^k \geq 0$, which together imply that the bracket is positive when $\mu^k(t) \leq 1$. Thus the correlation between the weight vectors increases during associative learning. Moreover, it can be checked similarly that $\left\langle w_i^k(t+\Delta t) w_i^k(t+\Delta t) \right\rangle = \left\langle w_i^k(t) v_i^k(t) \right\rangle = \left\langle v_i^k(t+\Delta t) v_i^k(t+\Delta t) \right\rangle = \left\langle v_i^k(t) v_i^k(t) \right\rangle = 1$, as long as it starts at 1 at $t = 0$. This justifies our choice of weight statistics [Eq. (S9)] as a description for the network during associative learning.

2.2. A Bayesian inference perspective of the network model

The predictive coding framework is often used to account for inference of latent causes of sensorimotor inputs to the brain, based on prediction and prediction-error signals [2–5]. In this section we show that our model can similarly be viewed as a network performing Bayesian inference. Specifically, the network's neural dynamics [Eq. (S4)] implement the inference (or state estimation) of latent variables driving inputs. Moreover, the slow synaptic weight changes during learning [Eq. (S9)] can be viewed as a mechanism for improving the accuracy of the inference performed by the network.

We consider a scenario where sensory inputs in the environment are generated by a probabilistic generative model, $p(\boldsymbol{x}, \boldsymbol{y}|\boldsymbol{r})$, where $\boldsymbol{x}, \boldsymbol{y}$ are the (possibly time-dependent) sensory inputs and $\boldsymbol{r}$ represents the latent variables that determine the statistics of the sensory inputs. We denote the prior distribution over the latent variables as $p_0(\boldsymbol{r})$. Then given the sensory inputs $\boldsymbol{x}, \boldsymbol{y}$, the latent variables $\boldsymbol{r}$ can be inferred by maximizing the posterior distribution via Bayes' rule,

$$p(\boldsymbol{r}|\boldsymbol{x}, \boldsymbol{y}) = \frac{p(\boldsymbol{x}, \boldsymbol{y}|\boldsymbol{r}) p_0(\boldsymbol{r})}{p(\boldsymbol{x}, \boldsymbol{y})}, \qquad \text{(S15)}$$

where $p(\boldsymbol{x}, \boldsymbol{y}) = \int p(\boldsymbol{x}, \boldsymbol{y}|\boldsymbol{r}) p_0(\boldsymbol{r}) d\boldsymbol{r}$ is the marginal distribution of the sensory inputs, independent of the latent variables.

Suppose that the generative distribution is a multivariate Gaussian and that its mean is a linear readout of the latent variables,

$$\ln p(\boldsymbol{x}, \boldsymbol{y}|\boldsymbol{r}) = -\frac{1}{\sigma_1^2} \sum_{k=1}^{P} \left[\left(x^k - \frac{1}{N} \boldsymbol{w}^k \cdot \boldsymbol{r} \right)^2 + \left(y^k - \frac{1}{N} \boldsymbol{v}^k \cdot \boldsymbol{r} \right)^2 \right] + \text{const.} \qquad \text{(S16)}$$

Further suppose that the prior distribution has the form,

$$\ln p_0(\boldsymbol{r}) = -\frac{1}{\sigma_0^2 N} \sum_{i=1}^{N} F(r_i) + \text{const.} \tag{S17}$$

Then, recalling Eq. (S7), we see that the neural dynamics [Eq. (S4)] maximize the log posterior distribution,

$$\ln p(\boldsymbol{r}|\boldsymbol{x}, \boldsymbol{y}) = \ln p(\boldsymbol{x}, \boldsymbol{y}|\boldsymbol{r}) + \ln p_0(\boldsymbol{r}) + \text{const} = -\frac{1}{\sigma_1^2} E(\boldsymbol{r}) + \text{const.} \tag{S18}$$

Here $E(\boldsymbol{r})$ is the objective function in the previous section with $b = \sigma_0^2/\sigma_1^2$. Thus, our model's gain parameter b is related to the prediction accuracy σ_1. The latent variables $\boldsymbol{r}$ here correspond to the firing rates of the neurons in the network.

In the more general case where sensory inputs are not generated exactly according to Eq. (S16), prediction accuracy can be improved by adjusting the readout weights $\boldsymbol{w}^k, \boldsymbol{v}^k$ to maximize the log posterior distribution [Eq. (S18)] based on the learning rule [Eq. (S11)]. This weight optimization procedure is equivalent to using a variational approach for maximizing the Bayesian model evidence, as introduced in previous predictive coding literature [4–6]. We also note that the nonlinear response function ϕ appears in the regularization $F(\boldsymbol{r})$ [Eq. (S5)] is linked to the 'encoding' of prior information on the latent variables, $p_0(\boldsymbol{r})$.

2.3. Extensions of the network model

2.3.1. Associations between more than two modalities

Our network model can be generalized to apply to scenarios in which the animal is trained to associate multiple ($M \geq 3$) sensorimotor inputs. Here the network generates internal predictions for each input, that can be linearly read-out,

$$\hat{x}_l^k(t) = \frac{1}{N} \boldsymbol{w}_l^k \cdot \boldsymbol{r}(t), \qquad k = 1, \ldots, P, \qquad l = 1, \ldots, M, \tag{S19}$$

where $\boldsymbol{w}_l^k$ are the readout weights for each input in each stimulus modality. The objective function [Eq. (S1)] is now,

$$E_M(t) = \sum_{l=1}^{M} \sum_{k=1}^{P} \left(x_l^k(t) - \hat{x}_l^k(t) \right)^2 + \frac{2}{b} \sum_{i=1}^{N} F(r_i(t)). \tag{S20}$$

The network dynamics and recurrent connectivity matrix are,

$$\tau \frac{\mathrm{d}h_i(t)}{\mathrm{d}t} = -h_i(t) + \sum_{j=1}^{N} J_{ij}^{M} \phi(h_j(t)) + \sum_{l=1}^{M} \sum_{k=1}^{P} b\, w_{l,i}^{k} x_l^{k},$$

$$J_{ij}^{M} = \frac{b}{N} \sum_{l=1}^{M} \sum_{k=1}^{P} w_{l,i}^{k} w_{l,j}^{k}. \tag{S21}$$

Using similar derivations as above, one can show that (*i*) $E_M(t)$ is a Lyapunov function for the network dynamics, and (*ii*) the network will reach a unique stable fixed point for any combination of the inputs x_l^k. Assuming that the feedforward weights corresponding to associated stimuli become increasingly correlated during learning (similarly to the $M = 2$ case), will make this model useful for studying predictive representations when training animals on more complex stimulus combinations.

2.3.2. Neurons with dendritic compartments

The network model with point neurons [Eq. (S4)] and the associated learning rules [Eq. (S11)] can be extended to a model with dendritic compartments. Crucially, this extension allows the learning rule to be realized by local plasticity rules.

Following the approach introduced in Refs. [7, 8], we first notice that Eqs. (S4-S5) can be rewritten by decomposing the connectivity to synaptic weights onto specific dendrites, giving,

$$J_{ij}^{k} = -\frac{b}{N} w_i^k w_j^k, \qquad J_{ij}^{k+P} = -\frac{b}{N} v_i^k v_j^k,$$

$$\tau \frac{\mathrm{d}h_i(t)}{\mathrm{d}t} = -h_i(t) + \sum_{k=1}^{P} \left[\sum_{j=1}^{N} J_{ij}^{k} \phi(h_j(t)) + w_i^k x^k \right] + \sum_{k=1}^{P'} \left[\sum_{j=1}^{N} J_{ij}^{k+P} \phi(h_j(t)) + v_i^k y^k \right]. \tag{S22}$$

Here we think of $h_i(t)$ as the somatic membrane potential of neuron i. Next we introduce $P + P'$ dendritic compartments corresponding to neuron i. The voltages u_i^k for $k = 1, \ldots, P$ and for $k = P+1, \ldots, P' + P$ are respectively governed by the equations,

$$\tau_u \frac{\mathrm{d}u_i^k(t)}{\mathrm{d}t} = -u_i^k(t) + \sum_{j=1}^{N} J_{ij}^{k} \phi(h_j(t)) + b w_i^k x^k,$$

$$\tau_u \frac{\mathrm{d}u_i^{k+P}(t)}{\mathrm{d}t} = -u_i^{k+P}(t) + \sum_{j=1}^{N} J_{ij}^{k+P} \phi(h_j(t)) + b v_i^k y^k. \tag{S23}$$

The somatic voltage level is then driven by the dendrites,

$$\tau \frac{\mathrm{d}h_i(t)}{\mathrm{d}t} = -h_i(t) + \sum_{k=1}^{P} u_i^k(t) + \sum_{k=1}^{P'} u_i^{k+P}(t). \tag{S24}$$

Under the assumption that dendrite voltage changes faster than somatic voltage, $\tau_u \ll \tau$, this recovers our original model with point neurons [Eq. (S4)].

The learning rule of the dendrite-specific feedforward weights is given by,

$$\Delta w_i^k = \frac{\eta}{N} \frac{x^k u_i^k r_i}{I_i^k}, \qquad \Delta v_i^k = \frac{\eta}{N} \frac{y^k u_i^{k+P} r_i}{I_i^{k+P}}, \tag{S25}$$

where we have denoted $I_i^k = bw_i^k x^k$ and $I_i^{k+P} = bv_i^k y^k$. Note that the quantities on the right hand side are 'local' to the feedforward synapses w_i^k and v_i^k. At steady-state, $u_i^k = bw_i^k \delta x^k$ and $u_i^{k+P} = bv_i^k \delta y^k$. Together with the definition of I_i^k, this learning rule is the same as Eq. (S10). To avoid unbounded growth of the weights in this setting, we assume a similar homeostatic mechanism which recovers the previous learning rule for the feedforward weights [Eq. (S11)].

The recurrent weights are subject to the learning rules,

$$\begin{aligned} \frac{\mathrm{d}J_{ij}^k}{\mathrm{d}t} &= -\frac{\eta}{N} u_i^k (r_j - \langle r_j \rangle) - \left[\frac{\eta_1^k}{I_i^k} (r_i - \langle r_i \rangle) + \eta_2^k \right] J_{ij}^k, \\ \frac{\mathrm{d}J_{ij}^{k+P}}{\mathrm{d}t} &= -\frac{\eta}{N} u_i^{k+P} (r_j - \langle r_j \rangle) - \left[\frac{\eta_1^{k+P}}{I_i^{k+P}} (r_i - \langle r_i \rangle) + \eta_2^{k+P} \right] J_{ij}^{k+P}, \end{aligned} \tag{S26}$$

where $\eta_1^k = \langle u_i^k \rangle$, $\eta_2^k = \langle u_i^k \rangle \hat{x}^k$ and $\eta_2^{k+P} = \langle u_i^{k+P} \rangle \hat{y}^k$ are activity-dependent learning rates. The dendrite-specific synaptic weights [Eq. (S22)] are solutions to these learning dynamics.

We note that the increase in correlation between $\boldsymbol{w}$ and $\boldsymbol{v}$ during learning is reflected in this plasticity rule by the dependence of both J^k and J^{k+P} on the firing rates $\boldsymbol{r}$. Since those rates depend on inputs from both modalities, both sets of dendrite-specific synaptic weights change based on the interplay between the multimodal input.

2.3.3. Hierarchical network architecture

In the recurrent network model studied thus far, a single module integrates inputs from multiple sensorimotor modalities. Here we generalize this model to a network consisting of multiple (L) modules arranged in a layered structure. Each module has N neurons with firing rates denoted as $\boldsymbol{r}^l$, $l = 1, \ldots, L$. We assume that the paired stimulus inputs enter the network via the first and the last module respectively (Fig. 7a). For convenience, we denote the inputs as $\boldsymbol{x} \equiv \boldsymbol{r}^0$ and $\boldsymbol{y} \equiv \boldsymbol{r}^{L+1}$.

Each module generates predictions of the activity of 'adjacent' (earlier and later) modules, i.e., neurons in module l generate predictions for neural responses in modules $l-1$ and $l+1$. Those predictions are assumed to be linear readouts of the firing rates,

$$\hat{\boldsymbol{r}}^{l-1} = \frac{1}{N} W^{l\top} \boldsymbol{r}^l, \qquad \hat{\boldsymbol{r}}^{l+1} = \frac{1}{N} V^{l\top} \boldsymbol{r}^l. \tag{S27}$$

Here W^l, V^l are the readout matrices. The objective function for this hierarchical network is a sum of the objective function applied to each module-separately with the corresponding prediction errors and firing-rate regularization,

$$\begin{aligned} \mathsf{E}(\{\boldsymbol{r}^l\}; \{W^l, V^l\}) &= \sum_{l=1}^{L} \left[\frac{1}{\sigma_l^2} (\boldsymbol{r}^{l-1} - \hat{\boldsymbol{r}}^{l-1})^2 + \frac{1}{\sigma_l^2} (\boldsymbol{r}^{l+1} - \hat{\boldsymbol{r}}^{l+1})^2 + \frac{F(\boldsymbol{r}^l)}{b_l} \right], \\ &= \sum_{l=1}^{L} \left[\frac{1}{\sigma_l^2} \left(\boldsymbol{r}^{l-1} - \frac{W^{l\top} \boldsymbol{r}^l}{N} \right)^2 + \frac{1}{\sigma_l^2} \left(\boldsymbol{r}^{l+1} - \frac{V^{l\top} \boldsymbol{r}^l}{N} \right)^2 + \frac{F(\boldsymbol{r}^l)}{b_l} \right], \\ &= \sum_{l=1}^{L} E(\boldsymbol{r}^l; W^l, V^l). \end{aligned} \tag{S28}$$

Here σ_l measures the module-specific precision of predictions and b_l is the module-specific regularization. The assumption that the neurons in module l minimize the module-specific loss $E(\boldsymbol{r}^l; W^l, V^l)$ implies that the neural dynamics within each module and the recurrent synaptic weights have identical form to those in the single-module case,

$$\begin{aligned} \sigma_l^2 \frac{\mathrm{d}h_i^l}{\mathrm{d}t} &= -\sigma_l^2 h_i^l(t) + \sum_{j=1}^{N} J_{ij}^l \phi(h_j^l(t)) + b_l \sum_{k=1}^{N} W_{ik}^l \phi(h_k^{l-1}(t)) + b_l \sum_{k'=1}^{N} V_{ik}^l \phi(h_k^{l+1}(t)), \\ J_{ij}^l &= -\frac{b_l}{N} \sum_{k=1}^{N} \left(W_{ik}^l W_{jk}^l + V_{ik}^l V_{jk}^l \right). \end{aligned} \tag{S29}$$

Similarly to the network with a single module, we assume that associative learning induces correlations between the corresponding weight vectors for each stimulus-pair. In the hierarchical network, the feedforward weight matrices in the first and last modules $W^{1\top}, V^1$ have dimensions $N \times P$ rather than the $N \times N$ dimensions of matrices in intermediate modules. We assume that the intermediate feedforward weight matrices have rank P (the stimulus dimension). Furthermore, because the process of training the network to associate stimuli x^k with y^k is symmetric under the substitutions $x \leftrightarrow y$, $W \leftrightarrow V$, we assume that W, V are symmetric matrices. With these assumptions, we the weight matrices are,

$$W^1 = \sum_{k=1}^{P} \hat{\boldsymbol{e}}_k (\boldsymbol{w}_k^1)^\top, \qquad W^l = \frac{1}{N} \sum_{k=1}^{P} \boldsymbol{w}_k^l (\boldsymbol{w}_k^l)^\top, \qquad\qquad l = 2, \ldots, L,$$

$$V^L = \sum_{k=1}^{P} \hat{\boldsymbol{e}}_k (\boldsymbol{v}_k^L)^\top, \qquad V^l = \frac{1}{N}\sum_{k=1}^{P} \boldsymbol{v}_k^l (\boldsymbol{v}_k^l)^\top, \qquad\qquad l = 1, \ldots, L-1. \tag{S30}$$

During associative learning, these weight vectors become correlated and their statistics are,

$$\begin{aligned} \langle w_{ki}^l \rangle = \langle v_{ki}^l \rangle = 0, \qquad \langle w_{ki}^l w_{k'j}^l \rangle = \langle v_{ki}^l v_{k'j}^l \rangle = \delta_{kk'}\delta_{ij}, \qquad \langle w_{ki}^l v_{k'j}^l \rangle = \delta_{kk'}\delta_{ij}\mu^k, \\ \langle w_{ki}^l w_{k'j}^{l'} \rangle = \langle v_{ki}^l v_{k'j}^{l'} \rangle = \langle w_{ki}^l v_{k'j}^{l'} \rangle = \delta_{kk'}\delta_{ij}\mu^k, \qquad l' \neq l. \end{aligned} \tag{S31}$$

Here the first line specifies the weight statistics within module l, and the second line specifies the statistics across modules. The recurrent connectivity within each module simplifies to a form which is identical to that of the single module network,

$$\begin{aligned} J^l &= -\frac{b_l}{N} \sum_{k,k'=1}^{P} \left[\boldsymbol{w}_{k'}^l \frac{\boldsymbol{w}_{k'}^{l\top} \boldsymbol{w}_k^l}{N} (\boldsymbol{w}_k^l)^\top + \boldsymbol{v}_{k'}^l \frac{\boldsymbol{v}_{k'}^{l\top} \boldsymbol{v}_k^l}{N} (\boldsymbol{v}_k^l)^\top \right] \\ &\overset{N\to\infty}{=} -\frac{b_l}{N} \sum_{k=1}^{P} \left[\boldsymbol{w}_k^l (\boldsymbol{w}_k^l)^\top + \boldsymbol{v}_k^l (\boldsymbol{v}_k^l)^\top \right]. \end{aligned} \tag{S32}$$

3. PREDICTIVE REPRESENTATIONS IN RECURRENT NETWORKS

When the stimulus inputs do not depend on time, the objective function E [Eq. (S2)] can be viewed as a function of the firing-rates and synaptic weights,

$$\begin{aligned} E(\boldsymbol{r}; \{\boldsymbol{w}^k, \boldsymbol{v}^k\}) &= \sum_{k=1}^{P} \left[\left(x^k - \frac{1}{N}\boldsymbol{w}^k \cdot \boldsymbol{r} \right)^2 + \left(y^k - \frac{1}{N}\boldsymbol{v}^k \cdot \boldsymbol{r} \right)^2 \right] + \frac{2}{bN}\sum_{i=1}^{N} F(r_i) \\ &= \frac{2}{bN} \left[\sum_{k=1}^{P} b \left(-x^k \boldsymbol{w}^k \cdot \boldsymbol{r} - y^k \boldsymbol{v}^k \cdot \boldsymbol{r} + \frac{(\boldsymbol{w}^k \cdot \boldsymbol{r})^2 + (\boldsymbol{v}^k \cdot \boldsymbol{r})^2}{2N} \right) + \sum_{i=1}^{N} F(r_i) \right] \\ &\quad + \sum_{i=1}^{P} \left[(x^k)^2 + (y^k)^2 \right]. \\ &\equiv \frac{2}{bN} E_0(\boldsymbol{r}; \{\boldsymbol{w}^k, \boldsymbol{v}^k\}) + \sum_{i=1}^{P} \left[(x^k)^2 + (y^k)^2 \right]. \end{aligned} \tag{S33}$$

The steady state firing-rates can be expressed as minimization over E_0, since the second term in Eq. (S33) does not depend on $\boldsymbol{r}$,

$$\boldsymbol{r}^\star = \underset{\boldsymbol{r} \in \mathbb{R}_+^n}{\operatorname{argmin}}\, E_0(\boldsymbol{r}; \{\boldsymbol{w}^k, \boldsymbol{v}^k\}). \tag{S34}$$

Next we will use the replica method [9, 10] to calculate the firing-rate distribution of neurons in the network,

$$p(r) = \frac{1}{N}\sum_{i=1}^{N}\delta(r - r_i). \tag{S35}$$

In general, firing-rates in the network depend on the specific realization of random weights $\boldsymbol{w}^k, \boldsymbol{v}^{k'}$. We find however that in the $N \to \infty$ limit, the firing-rate distribution is self-averaging and depends only on the distribution of synaptic weights. By choosing which of the x^k and y^k's are nonzero, we can study the network response in different stimulus conditions. For convenience, we assume that at any given time, only a finite number of stimulus-pairs are presented, or equivalently, there are only $K = O(1)$ pairs (x^k, y^k) for $k = 1, \ldots, K$, where at least one stimulus is nonzero. We set the decay timescale to $\tau = 1$.

3.1. Replica calculation of the firing-rate statistics

We consider the partition function

$$Z = \int_{\mathbb{R}_+^N} e^{-\beta E_0(\boldsymbol{r};\{\boldsymbol{w}^k,\boldsymbol{v}^k\})}\,\mathrm{d}\boldsymbol{r}. \tag{S36}$$

We suppress the domain of integration over firing-rates for readability in the following calculations. In the limit $\beta \to \infty$, the dominant contribution to Z comes from the fixed point solution which minimizes $E_0(\boldsymbol{r}; \{\boldsymbol{w}^k, \boldsymbol{v}^k\})$ in Eq. (S34). The logarithm of the partition function concentrates around its expectation, so we use the replica trick,

$$\lim_{N\to\infty}\frac{\ln Z}{N} = \lim_{N\to\infty}\left\langle \frac{\ln Z}{N}\right\rangle = \lim_{n\to 0}\lim_{N\to\infty}\frac{\ln\langle Z^n\rangle}{nN}. \tag{S37}$$

We make the standard assumption that the order of the limits can be exchanged in the last equality. We first calculate $\langle Z^n\rangle$. For readability, we use g for the gain parameter (instead of b) in Subsection 3.1, and $a, b = 1, \ldots, n$ for the replica indices. Without loss of generality, we assume that the presented stimuli (i.e., indices k such that x_k or y_k is nonzero) are the first K pairs, $k = 1, \ldots, K$.

$$\begin{aligned}
\langle Z^n\rangle = &\int \prod_a \mathrm{d}\boldsymbol{r}^a \left\langle \exp\left\{-\beta\sum_{i,a}F(r_i^{\alpha}) - \frac{g\beta}{2N}\sum_a\sum_{k=1}^{P}\left[(\boldsymbol{w}^k\cdot\boldsymbol{r}^a)^2 + (\boldsymbol{v}^k\cdot\boldsymbol{r}^a)^2\right]\right\}\right. \\
&\left.\times \exp\left[g\beta\sum_a\sum_{s=1}^{K}(x^s\boldsymbol{w}^s\cdot\boldsymbol{r}^a + y^s\boldsymbol{v}^s\cdot\boldsymbol{r}^a)\right]\right\rangle, \\
= &\int\prod_{a,i}\mathrm{d}r_i^a \left\langle \exp\left\{-\beta\sum_{i,a}F(r_i^a) - \frac{g\beta}{2N}\sum_a\sum_{k=K+1}^{P}\left[(\boldsymbol{w}^k\cdot\boldsymbol{r}^a)^2 + (\boldsymbol{v}^k\cdot\boldsymbol{r}^a)^2\right]\right\}\right\rangle
\end{aligned}$$

$$
\times \left\langle \exp\left\{ g\beta \sum_a \sum_{k=1}^{K} \left[\left(x^k \boldsymbol{w}^k \cdot \boldsymbol{r}^a + y^k \boldsymbol{v}^k \cdot \boldsymbol{r}^a \right) - \frac{1}{2N} \left((\boldsymbol{w}^k \cdot \boldsymbol{r}^a)^2 + (\boldsymbol{v}^k \cdot \boldsymbol{r}^a)^2 \right) \right] \right\} \right\rangle
$$
$$
= \int \prod_{a,i} \mathrm{d}r_i^a \exp\left[-\beta \sum_{a,i} F(r_i^a) \right] \left\langle \exp\left\{ -\frac{g\beta}{2N} \sum_a \sum_{k=K+1}^{P} \left[(\boldsymbol{w}^k \cdot \boldsymbol{r}^a)^2 + (\boldsymbol{v}^k \cdot \boldsymbol{r}^a)^2 \right] \right\} \right\rangle
$$
$$
\times \left\langle \exp\left\{ g\beta \sum_a \sum_{k=1}^{K} \left[-\frac{1}{2N} (\boldsymbol{w}^k \cdot \boldsymbol{r}^a)^2 - \frac{1}{2N} (\boldsymbol{v}^k \cdot \boldsymbol{r}^a)^2 + x^k \boldsymbol{w}^k \cdot \boldsymbol{r}^a + y^k \boldsymbol{v}^k \cdot \boldsymbol{r}^a \right] \right\} \right\rangle . \tag{S38}
$$

Notice that we have split the summation over all P stimulus-pairs and averaging over the corresponding synaptic weights into the presented pairs ($k = 1, \ldots, K$) and the rest ($k = K+1, \ldots, P$). We first perform calculations for the $P - K$ 'absent' stimulus-pairs. Using the integral representation of Gaussian function, we get,

$$
e^{-\frac{g\beta}{2N}(\boldsymbol{w}^k \cdot \boldsymbol{r}^a)^2} = \int \frac{\mathrm{d}t^{k,a}}{\sqrt{2\pi}} \sqrt{g\beta} e^{-g\beta\left[\frac{(t^{k,a})^2}{2} + i t^{k,a} \frac{\boldsymbol{w}^k \cdot \boldsymbol{r}}{\sqrt{N}} \right]},
$$
$$
e^{-\frac{g\beta}{2N}(\boldsymbol{v}^k \cdot \boldsymbol{r}^a)^2} = \int \frac{\mathrm{d}s^{k,\alpha}}{\sqrt{2\pi}} \sqrt{g\beta} e^{-g\beta\left[\frac{(s^{k,a})^2}{2} + i s^{k,a} \frac{\boldsymbol{v}^k \cdot \boldsymbol{r}}{\sqrt{N}} \right]}. \tag{S39}
$$

Using these, the term corresponding to the $P - K$ absent stimulus-pairs becomes,

$$
\left\langle \exp\left\{ -\frac{g\beta}{2N} \sum_a \sum_{k=K+1}^{P} \left[(\boldsymbol{w}^k \cdot \boldsymbol{r}^a)^2 + (\boldsymbol{v}^k \cdot \boldsymbol{r}^a)^2 \right] \right\} \right\rangle
$$
$$
= \left\langle \prod_a \prod_{k=K+1}^{P} \frac{g\beta}{2\pi} \int \mathrm{d}t^{k,a} \mathrm{d}s^{k,a} e^{-g\beta\left[\frac{(t^{k,a})^2 + (s^{k,a})^2}{2} + \frac{i}{\sqrt{N}} (t^{k,a} \boldsymbol{w}^k \cdot \boldsymbol{r}^a + s^{k,a} \boldsymbol{v}^k \cdot \boldsymbol{r}^a) \right]} \right\rangle
$$
$$
= \prod_{k=K+1}^{P} \left(\frac{g\beta}{2\pi} \right)^n \int \prod_a \mathrm{d}t^{k,a} \mathrm{d}s^{k,a} e^{-\frac{g\beta}{2} \sum_a \left[(t^{k,a})^2 + (s^{k,a})^2 \right]} \left\langle e^{-\frac{ig\beta}{\sqrt{N}} \sum_a (t^{k,a} \boldsymbol{w}^k \cdot \boldsymbol{r}^a + s^{k,a} \boldsymbol{v}^k \cdot \boldsymbol{r}^a)} \right\rangle
$$
$$
= \prod_{k=K+1}^{P} \left[\left(\frac{g\beta}{2\pi} \right)^n \int \prod_\alpha \mathrm{d}t^a \mathrm{d}s^a e^{-\frac{g\beta}{2} \sum_a \left[(t^a)^2 + (s^a)^2 \right]} \left\langle e^{-\frac{ig\beta}{\sqrt{N}} \left[(\sum_a t^a \boldsymbol{r}^a) \cdot \boldsymbol{w} + (\sum_a s^a \boldsymbol{r}) \cdot \boldsymbol{v} \right]} \right\rangle \right]. \tag{S40}
$$

In the last line we have suppressed the superscript k. Recall that for each k, angle brackets denote the average over a pair of synaptic weight vectors, each of which has components sampled from the same distribution with mean 0 and correlation μ^k [Eq. (S9)]. We work out the last factor of the integrand,

$$
\left\langle e^{-\frac{ig\beta}{\sqrt{N}} \left[(\sum_a t^a \boldsymbol{r}^a) \cdot \boldsymbol{w} + (\sum_a s^a \boldsymbol{r}^a) \cdot \boldsymbol{v} \right]} \right\rangle = \left\langle \prod_{j=1}^{N} e^{-\frac{ig\beta}{\sqrt{N}} \left[(\sum_a t^a r_j^a) w_j + (\sum_a s^a r_j^a) v_j \right]} \right\rangle
$$

$$= \prod_{j=1}^{N} f\left(-\frac{g\beta}{\sqrt{N}}\sum_a t^a r_j^a\,,\ -\frac{g\beta}{\sqrt{N}}\sum_a s^a r_j^a\right), \tag{S41}$$

where $f(x, y)$ is the joint characteristic function of the random vectors $\boldsymbol{w}^k, \boldsymbol{v}^k$ with correlation μ^k. The Taylor expansion of $f(\cdot,\cdot)$ in the limit $N \to \infty$ is,

$$f\left(-\frac{g\beta}{\sqrt{N}}\sum_a t^a r_j^a\,,\ -\frac{g\beta}{\sqrt{N}}\sum_a s^a r_j^a\right)$$
$$= 1 - \frac{g^2\beta^2}{2N}\left[\left(\sum_a t^a r_j^a\right)^2 + 2\mu^k\left(\sum_a t^a r_j^a\right)\left(\sum_a s^a r_j^a\right) + \left(\sum_a s^a r_j^a\right)^2\right] + O(N^{-2}). \tag{S42}$$

Using this we get,

$$\left\langle e^{-\frac{ig\beta}{\sqrt{N}}\left[(\sum_a t^a \boldsymbol{r}^a)\cdot\boldsymbol{w} + (\sum_a s^a \boldsymbol{r}^a)\cdot\boldsymbol{v}\right]}\right\rangle$$
$$\underset{N\to\infty}{\longrightarrow} \prod_{j=1}^{N}\left[1 - \frac{g\beta}{2N}\left[\left(\sum_a t^a r_j^a\right)^2 + 2\mu^k\left(\sum_a t^a r_j^a\right)\left(\sum_a s^a r_j^a\right) + \left(\sum_a s^a r_j^a\right)^2\right]\right]$$
$$\underset{e^x\approx 1+x}{\longrightarrow} \exp\left[-\frac{g\beta}{2}\sum_{a,b}\left(\frac{\sum_{j=1}^{N} r_j^a r_j^b}{N}\right)\left(t^a t^b + 2\mu^k t^a s^b + s^a s^b\right)\right]$$
$$= \exp\left[-\frac{g\beta}{2}\sum_{a,b} q^{ab}\left(t^a t^b + 2\mu^k t^a s^b + s^a s^b\right)\right]. \tag{S43}$$

In the last line we have introduced the usual definition of the order parameter,

$$q^{ab} = \frac{1}{N}\sum_{j=1}^{N} r_j^a r_j^b. \tag{S44}$$

Collecting terms, we find that Eq. (S40) becomes,

$$\left\langle \exp\left\{-\frac{g\beta}{2N}\sum_a \sum_{k=K+1}^{P}\left[(\boldsymbol{w}^k\cdot\boldsymbol{r}^a)^2 + (\boldsymbol{v}^k\cdot\boldsymbol{r}^a)^2\right]\right\}\right\rangle$$
$$= \prod_{k=K+1}^{P}\left\{\left(\frac{g\beta}{2\pi}\right)^n \int \prod_a \mathrm{d}t^a \mathrm{d}s^a\right.$$
$$\left.\times \exp\left[-\frac{g\beta}{2}\left(\sum_a\left[(t^a)^2 + (s^a)^2\right] + g\beta\sum_{a,b} q^{ab}\left(t^a t^b + 2\mu^k t^a s^b + s^a s^b\right)\right)\right]\right\}$$

$$
= \prod_{k=K+1}^{P} \left\{ \left(\frac{1}{2\pi}\right)^n \int \mathrm{d}\boldsymbol{t}\mathrm{d}\boldsymbol{s} \exp\left[-\frac{1}{2} \begin{pmatrix} \boldsymbol{t} \\ \boldsymbol{s} \end{pmatrix}^\top \begin{pmatrix} I_n + g\beta q & \mu^k g\beta q \\ \mu^k g\beta q & I_n + g\beta q \end{pmatrix} \begin{pmatrix} \boldsymbol{t} \\ \boldsymbol{s} \end{pmatrix} \right] \right\}
$$

$$
= \sqrt{\prod_{k=K+1}^{P} \det \begin{pmatrix} I_n + g\beta q & \mu^k g\beta q \\ \mu^k g\beta q & I_n + g\beta q \end{pmatrix}^{-1}}, \tag{S45}
$$

Here q is an $n \times n$ matrix [Eq. (S44)] and I_n is the $n \times n$ identity matrix. In the next to last step of Eq. (S45) we rescaled the integration variables t, s by $\sqrt{g\beta}$.

The term in Eq. (S38) corresponding to the K presented pairs can be calculated in a similar fashion, which yields,

$$
\left\langle \exp\left\{ g\beta \sum_a \sum_{k=1}^{K} \left[-\frac{1}{2N}(\boldsymbol{w}^k \cdot \boldsymbol{r}^a)^2 - \frac{1}{2N}(\boldsymbol{v}^k \cdot \boldsymbol{r}^a)^2 + x^k \boldsymbol{w}^k \cdot \boldsymbol{r}^a + y^k \boldsymbol{v}^k \cdot \boldsymbol{r}^a \right] \right\} \right\rangle
$$

$$
= \int (g\beta)^{nK} \prod_a \prod_{k=1}^{K} \frac{\mathrm{d}t^{k,a}\mathrm{d}s^{k,a}}{2\pi} e^{+\frac{g\beta N}{2}\sum_{k,a}\left[(t^{k,a})^2 + (s^{k,a})^2\right]} \left\langle e^{g\beta \sum_{k,a,i}\left[(x^k t^{k,\alpha}) w_i^k r_i^a + (y^k - s^{k,a}) v_i^k r_i^a\right]} \right\rangle . \tag{S46}
$$

We introduce the delta function to enforce the definition of the order parameter q,

$$
\delta\left(q^{ab} - \frac{1}{N}\sum_{j=1}^{N} r_j^a r_j^b \right) = N \int \frac{\mathrm{d}\hat{q}^{ab}}{2\pi} e^{q^{ab}(N\hat{q}^{ab} - \sum_j r_j^a r_j^b)}. \tag{S47}
$$

Putting all terms together Eq. (S38) gives,

$$
\begin{aligned}
\langle Z^n \rangle =& (g\beta)^{nP} N^{\frac{n^2}{2}} \int \prod_{k,a} \frac{\mathrm{d}t^{k,a}\mathrm{d}s^{k,a}}{2\pi} \prod_{a,b} \frac{\mathrm{d}\hat{q}^{ab}\mathrm{d}q^{ab}}{2\pi} e^{N\sum_{a,b}\hat{q}^{ab}\hat{q}^{ab} - \frac{g\beta N}{2}\sum_{k,a}[(t^{k,a})^2 + (s^{k,a})^2]} \\
&\times \left\{ \int \prod_a \mathrm{d}r^a \left\langle e^{-\beta\sum_a F(r^a) + g\beta\sum_{k,a}\left[(x^k - it^{k,a})w^k r^a + (y^k - is^{k,a})v^k r^a\right] - \sum_{a,b}\hat{q}^{ab} r^a r^b} \right\rangle \right\}^N \\
&\times \prod_{k=K+1}^{P} \sqrt{\det \begin{pmatrix} I_n + g\beta q & \mu^k g\beta q \\ \mu^k g\beta q & I_n + g\beta q \end{pmatrix}^{-1}} \\
=& \int \prod_{k,\alpha} \frac{\mathrm{d}t^{k,a}\mathrm{d}s^{k,a}}{2\pi} \prod_{a,b} \frac{\mathrm{d}\hat{q}^{ab}\mathrm{d}q^{ab}}{2\pi} e^{N\mathcal{F}(q^{ab},\hat{q}^{ab},t^{k,a},s^{k,a})}.
\end{aligned} \tag{S48}
$$

In the last line we have defined $\mathcal{F}(q^{ab}, \hat{q}^{ab}, t^{k,a}, s^{k,a})$ as,

$$
\mathcal{F}(q^{ab}, \hat{q}^{ab}, t^{k,a}, s^{k,a}) =
$$

$$
\begin{aligned}
&\frac{nK}{N}\ln(g\beta)+\frac{n^2}{2}\frac{\ln N}{N}+\sum_{a,b}q^{ab}\hat{q}^{ab}-\frac{g\beta}{2}\sum_{a,k}[(t^{a,k})^2+(s^{a,k})^2]\\
&+\ln\left\{\int\prod_a \mathrm{d}r^a\left\langle e^{-\beta\sum_a F(r^a)+g\beta\sum_{k,a}\left[(x^k-it^{k,a})w^k r^a+(y^k-is^{k,a})v^k r^a\right]-\sum_{a,b}\hat{q}^{ab}r^a r^b}\right\rangle\right\}\\
&-\frac{1}{2N}\sum_{k=K+1}^{P}\ln\det\begin{pmatrix} I_n+g\beta q & \mu^k g\beta q\\ \mu^k g\beta q & I_n+g\beta q\end{pmatrix}.
\end{aligned}
\tag{S49}
$$

In the limit $N\to\infty$, we use the saddle point approximation to compute the integral in the last line of Eq. (S48). Furthermore, because the Lyapunov function E_0 is convex [Eq. (S33)], the saddle point solution is replica symmetric, i.e.,

$$
q^{ab}=q_0\delta_{ab}+q_1(1-\delta_{ab}),\qquad \hat{q}^{ab}=\hat{q}_0\delta_{ab}+\hat{q}_1(1-\delta_{ab}),\qquad t^{a,k}=t^k,\qquad s^{a,k}=s^k. \tag{S50}
$$

We then simplify the terms in $\mathcal{F}$,

$$
\begin{aligned}
\sum_{a,b}q^{ab}\hat{q}^{ab}&=nq_0\hat{q}_0+n(n-1)q_1\hat{q}_1,\\
\sum_{a,k}[(t^{a,k})^2+(s^{a,k})^2]&=n\sum_k[(t^k)^2+(s^k)^2].
\end{aligned}
\tag{S51}
$$

$$
\begin{aligned}
\ln\det\begin{pmatrix} I_n+g\beta q & \mu^k g\beta q\\ \mu^k g\beta q & I_n+g\beta q\end{pmatrix}&=\ln\det[I_n+g\beta(1-\mu^k)q]+\ln\det[I_n+g\beta(1+\mu^k)q]\\
&=n\ln\left[(1+g\beta(1-\mu^k)(q_0-q_1))(1+g\beta(1+\mu^k)(q_0-q_1))\right]\\
&\quad+\ln\left[\left(1+\frac{g\beta(1-\mu^k)nq_1}{1+g\beta(1-\mu^k)(q_0-q_1)}\right)\left(1+\frac{g\beta(1+\mu^k)nq_1}{1+g\beta(1+\mu^k)(q_0-q_1)}\right)\right].
\end{aligned}
$$

Simplifying the term in the third line of Eq. (S49) requires a number of additional steps. Using the integral representation of Gaussian function we write,

$$
\begin{aligned}
e^{-\sum_{a,b}\hat{q}^{ab}r^a r^b}&=e^{-(\hat{q}_0-\hat{q}_1)\sum_a(r^a)^2-\hat{q}_1(\sum_a r^a)^2}\\
&=e^{-(\hat{q}_0-\hat{q}_1)\sum_a(r^a)^2}\int\frac{\mathrm{d}z}{\sqrt{2\pi}}e^{-\frac{z^2}{2}+i\sum_a\sqrt{2\hat{q}_1}r^a z}.
\end{aligned}
\tag{S52}
$$

Substituting this into the integral in Eq. (S49) gives,

$$
\begin{aligned}
&\int\prod_a \mathrm{d}r^a\left\langle e^{-\beta\sum_a F(r^a)+g\beta\sum_{k,a}\left[(x^k-it^{k,a})w^k r^a+(y^k-is^{k,a})v^k r^a\right]-\sum_{a,b}\hat{q}^{ab}r^a r^b}\right\rangle\\
&=\int\frac{\mathrm{d}z}{\sqrt{2\pi}}e^{-\frac{z^2}{2}}\prod_a\left[\int \mathrm{d}\nu_\beta(r^a)e^{-(\hat{q}_0-\hat{q}_1)(r^a)^2+i\sqrt{2\hat{q}}r^a z}\left\langle e^{g\beta(x^k-it^{k,a})w^k r^a+(y^k-is^{k,a})v^k r^a}\right\rangle\right]
\end{aligned}
$$

$$= \int Dz \left[\int \mathrm{d}\nu_\beta(r) e^{-(\hat{q}_0 - \hat{q}_1) r^2 + i\sqrt{2\hat{q}} rz} \left\langle e^{g\beta(x^k - it^k) w^k r + (y^k - is^k) v^k r} \right\rangle \right]^n. \tag{S53}$$

Here we have introduced the notation,

$$Dz = \frac{\mathrm{d}z}{\sqrt{2\pi}} e^{-\frac{z^2}{2}}, \qquad \mathrm{d}\nu_\beta(r) = \mathrm{d}r\, e^{-\beta F(r)}. \tag{S54}$$

Therefore, under the replica symmetric ansatz, Eq. (S49) becomes

$$\begin{aligned}
&\mathcal{F}(q_0, q_1, \hat{q}_0, \hat{q}_1, t^k, s^k) = \\
&\quad \frac{n^2}{2} \frac{\ln N}{N} + n q_0 \hat{q}_0 + n(n-1) q_1 \hat{q}_1 - \frac{ng\beta}{2} \sum_k [(t^k)^2 + (s^k)^2] \\
&\quad + \ln \int Dz \left[\int \mathrm{d}\nu_\beta(r) e^{-(\hat{q}_0 - \hat{q}_1) r^2 + i\sqrt{2\hat{q}_1} rz} \left\langle \prod_k e^{b\beta(x^k - it^k) w^k r + (y^k - is^k) v^k r} \right\rangle \right]^n \\
&\quad - \frac{1}{2N} \sum_{k=K+1}^{P} \Bigg\{ n \ln \left[(1 + g\beta(1-\mu^k)(q_0 - q_1))(1 + g\beta(1+\mu^k)(q_0 - q_1)) \right] \\
&\qquad + \ln \left[\left(1 + \frac{g\beta(1-\mu^k) n q_1}{1 + g\beta(1-\mu^k)(q_0 - q_1)} \right) \left(1 + \frac{g\beta(1+\mu^k) n q_1}{1 + g\beta(1+\mu^k)(q_0 - q_1)} \right) \right] \Bigg\}.
\end{aligned} \tag{S55}$$

Now we take the limits $P, N \to \infty$ and $n \to 0$, and identify $\alpha = P/N$, which gives,

$$\begin{aligned}
\lim_{\substack{N\to\infty \\ n\to 0}} \frac{\ln \langle Z^n \rangle}{nN} &= \lim_{\substack{N\to\infty \\ n\to 0}} \frac{\mathcal{F}(q_0, q_1, \hat{q}_0, \hat{q}_1, t^k, s^k)}{n} \\
&= q_0 \hat{q}_0 - q_1 \hat{q}_1 - g\beta \sum_k \frac{(t^k)^2 + (s^k)^2}{2} \\
&\quad + \int Dz \ln \int \mathrm{d}\nu_\beta(r) e^{-(\hat{q}_0 - \hat{q}_1) r^2 + i\sqrt{2\hat{q}_1} rz} \left\langle \prod_k e^{g\beta(x^k - it^k) w^k r + (y^k - is^k) v^k r} \right\rangle \\
&\quad - \frac{\alpha}{2} \Bigg\langle \ln \left[(1 + g\beta(1-\mu^k)(q_0 - q_1))(1 + g\beta(1+\mu^k)(q_0 - q_1)) \right] \\
&\qquad + \frac{g\beta(1-\mu^k) q_1}{1 + g\beta(1-\mu^k)(q_0 - q_1)} + \frac{g\beta(1+\mu^k) q_1}{1 + g\beta(1+\mu^k)(q_0 - q_1)} \Bigg\rangle_\mu.
\end{aligned} \tag{S56}$$

In the third line of Eq. (S56) we used the fact that for a well behaved function $A(z)$,

$$\lim_{n\to 0} \frac{1}{n} \ln \int Dz A^n(z) = \int Dz \ln A(z). \tag{S57}$$

The last term in Eq. (S56) (proportional to $\alpha/2$) was obtained by taking the limit over $P, N \to \infty$ and introducing α, and assuming that the number of presented stimulus-pairs

K is finite (necessary for the neural activity to remain finite, justified below). The average $\langle \cdots \rangle_\mu$ is over the distribution of correlation values μ^k, $k = 1, \ldots, P$ (i.e., over all learned stimulus-pairs).

To simplify the calculations, we define new variables

$$q' = g\beta(q_0 - q_1), \qquad \hat{q}' = 2\frac{\hat{q}_0 - \hat{q}_1}{\alpha g\beta}, \qquad \hat{q} = -\frac{2\hat{q}_1}{\alpha g^2\beta^2}, \qquad q = \frac{q_1}{g\beta}. \tag{S58}$$

and further make the change of variables, $t^k \to it^k$ and $s^k \to is^k$. With these simplifications, we rewrite Eq. (S56) as,

$$\begin{aligned}
&\lim_{\substack{N\to\infty \\ n\to 0}} \frac{\mathcal{F}(q', q, \hat{q}', \hat{q}, t^k, s^k)}{n} = \\
&\quad \frac{\alpha g\beta}{2}\left(\hat{q}'q - \hat{q}q'\right) + \alpha\frac{\hat{q}'q'}{2} + g\beta\sum_k \frac{(t^k)^2 + (s^k)^2}{2} \\
&\quad + \int Dz \ln\left[\int \mathrm{d}\nu_\beta(r) e^{-g\beta\frac{\alpha\hat{q}'r^2}{2} + g\beta\sqrt{\alpha\hat{q}}rz}\left\langle e^{g\beta r\sum_k\left[(x^k - t^k)w^k + (y^k - s^k)v^k\right]}\right\rangle\right] \\
&\quad - \frac{\alpha}{2}\left\langle \ln(1 + (1-\mu)q')(1 + (1+\mu)q') + \frac{(1-\mu)g\beta q}{1 + (1-\mu)q'} + \frac{(1+\mu)g\beta q}{1 + (1+\mu)q'}\right\rangle_\mu .
\end{aligned} \tag{S59}$$

To extract information about the network's response properties as $N \to \infty$, we evaluated these expressions at the saddle point of $\mathcal{F}(q_0, q_1, \hat{q}_0, \hat{q}_1, t^k, s^k)$. The saddle point satisfies,

$$\begin{aligned}
0 &= \frac{\partial\mathcal{F}}{\partial t^k} = g\beta\left(t^k - \int \mathrm{d}Q_\beta\, w^k r\right), \\
0 &= \frac{\partial\mathcal{F}}{\partial s^k} = g\beta\left(s^k - \int \mathrm{d}Q_\beta v^k r\right), \\
0 &= \frac{\partial\mathcal{F}}{\partial q} = \frac{\alpha g\beta}{2}\left[\hat{q}' - \left\langle \frac{1-\mu}{1 + (1-\mu)q'} + \frac{1+\mu}{1 + (1+\mu)q'}\right\rangle_\mu\right], \\
0 &= \frac{\partial\mathcal{F}}{\partial q'} = \frac{\alpha g\beta}{2}\left[\frac{\hat{q}'}{g\beta} - \hat{q} + \left\langle \frac{(1-\mu)^2}{[1 + (1-\mu)q']^2}q + \frac{(1+\mu)^2}{[1 + (1+\mu)q']^2}q\right\rangle_\mu\right], \\
0 &= \frac{\partial\mathcal{F}}{\partial \hat{q}'} = \frac{\alpha g\beta}{2}\left(\frac{q'}{g\beta} + q - \int \mathrm{d}Q_\beta r^2\right), \\
0 &= \frac{\partial\mathcal{F}}{\partial \hat{q}} = \frac{\alpha g\beta}{2}\left(-q' + \frac{1}{\sqrt{\alpha\hat{q}}}\int \mathrm{d}Q_\beta\, rz\right).
\end{aligned} \tag{S60}$$

Here we have defined the probability measure $\mathrm{d}Q_\beta$ as,

$$\left\langle \int \mathrm{d}Q_\beta(\ldots)\right\rangle = \int Dz \frac{\int \mathrm{d}\nu_\beta(r) e^{-g\beta\frac{\alpha\hat{q}'r^2}{2} + g\beta\sqrt{\alpha\hat{q}}rz}\left\langle e^{g\beta r\sum_k\left[(x^k - t^k)w^k + (y^k - s^k)v^k\right]}(\ldots)\right\rangle}{\int \mathrm{d}\nu_\beta(r) e^{-g\beta\frac{\alpha\hat{q}'r^2}{2} + g\beta\sqrt{\alpha\hat{q}}rz}\left\langle e^{g\beta r\sum_k\left[(x^k - t^k)w^k + (y^k - s^k)v^k\right]}\right\rangle}. \tag{S61}$$

Indeed, the probability measure $\mathrm{d}Q_\beta$ contains a Boltzmann distribution with the corresponding Hamiltonian,

$$\mathcal{H}(r) = F(r) + \frac{g\alpha\hat{q}' r^2}{2} - g\sqrt{\alpha\hat{q}}zr - gr\sum_{k=1}^{K}\left[(x^k - t^k)w^k + (y^k - s^k)v^k\right]. \tag{S62}$$

In the limit $\beta \to \infty$ ('zero temperature'), the Boltzmann distribution is dominated by the minimum of $\mathcal{H}(r)$ (i.e., the 'ground-state'). Since $\mathcal{H}(r)$ is strictly convex, there is a unique minimum $r^\star \geq 0$.

If $r^\star > 0$, the ground state satisfies $\mathcal{H}'(r^\star) = 0$, or equivalently,

$$0 = \phi_+^{-1}(r^\star) + \theta + g\alpha\hat{q}' r^\star - g\sqrt{\alpha\hat{q}}z - g\sum_{k=1}^{K}\left[(x^k - t^k)w^k + (y^k - s^k)v^k\right]. \tag{S63}$$

Otherwise, $r^\star = 0$. Indeed, the two cases can be written in a compact way,

$$r^\star = \phi\left(-g\alpha\hat{q}' r^\star + g\sqrt{\alpha\hat{q}}z + g\sum_{k=1}^{K}\left[(x^k - t^k)w^k + (y^k - s^k)v^k)\right]\right). \tag{S64}$$

The solution of above equation defines a function $r^\star(w^k, v^k, z)$. We recognize the argument of ϕ as the total input to each neuron and $r^\star$ as its nonlinear firing-rate response. It is important to note that the solution $r^\star$ depends on the Gaussian integration variable (z), the random synaptic weights (w, v), and the variables indicating the stimuli being presented (x, y), so overall the saddle point equations are expected to give a distribution of firing-rates, not a single value.

Substituting the ground-state solution $r^\star$ into the saddle point equation, we get at $\beta \to \infty$,

$$\begin{aligned}
t^k &= \left\langle w^k r^\star(w^k, v^k, z)\right\rangle_{w^k, v^k, z}, \\
s^k &= \left\langle v^k r^\star(w^k, v^k, z)\right\rangle_{w^k, v^k, z}, \\
\hat{q}' &= \left\langle \frac{1-\mu}{1+(1-\mu)q'}\right\rangle_\mu + \left\langle \frac{1+\mu}{1+(1+\mu)q'}\right\rangle_\mu, \\
\hat{q} &= \left\langle \left[\frac{1-\mu}{1+(1-\mu)q'}\right]^2\right\rangle_\mu q + \left\langle \left[\frac{1+\mu}{1+(1+\mu)q'}\right]^2\right\rangle_\mu q, \\
q &= \left\langle r^\star(w^k, v^k, z)^2\right\rangle_{w^k, v^k, z}, \\
q' &= \frac{1}{\sqrt{\alpha\hat{q}_1}}\left\langle r^\star(w^k, v^k, z)z\right\rangle_{w^k, v^k, z}.
\end{aligned} \tag{S65}$$

Notice that the order parameters t^k and s^k coincide with the internal predictions $\hat{x}^k$ and $\hat{y}^k$ [Eq. (S1)], and the order parameter q is the second moment of the firing-rate distribution.

3.2. Single-neuron and population statistics

We summarize the main results obtained from the above calculations: Given the distribution of synaptic weights $\{w^k, v^k\}$ and a standard normal random variable z, the firing-rate distribution $p(r)$ is the same as the distribution of the ground-state firing-rate $r^\star(w^k, v^k, z)$ [Eq. (S64)]. The order parameters $q, q', \hat{q}, \hat{q}', t^k = \hat{x}^k, s^k = \hat{y}^k$ which appear in $r^\star(w^k, v^k, z)$ need to be solved from the saddle point equations [Eq. (S60)]. Moreover, the voltage distribution of the neurons in the network is simply the distribution of the argument of the firing-rate transfer function ϕ in Eq. (S64), i.e.,

$$h^\star(w^k, v^k, z) \equiv -b\alpha\hat{q}' r^\star(w^k, v^k, z) + b\sqrt{\alpha\hat{q}}z + b\sum_{k=1}^{K}\left[(x^k - t^k)w^k + (y^k - s^k)v^k)\right]. \quad \text{(S66)}$$

Below we restrict our analysis to the special case where $\{w^k, v^k\}$ follow a multivariate Gaussian distribution; all the stimulus-pairs are learned equally well $\mu^k = \mu$; and the activation function is ReLU, $\phi = [x - \theta]_+$.

3.2.1. The high-dimensional case, $P/N \to \alpha > 0$

Under the above assumptions, Eq. (S34) can be solved exactly, giving neurons' firing-rate and voltage distributions,

$$\begin{aligned} r^\star(w^k, v^k, z) &= \frac{b}{1+\alpha b\hat{q}'}\left[\sqrt{\alpha\hat{q}}z + \sum_{k=1}^{K}\left[(x^k - t^k)w^k + (y^k - s^k)v^k)\right] - \frac{\theta}{b}\right]_+, \\ &\equiv \frac{b}{1+\alpha\hat{q}'b}\left[I - \frac{\theta}{b}\right]_+. \\ h^\star(w^k, v^k, z) &= I - \frac{\alpha\hat{q}'b}{1+\alpha\hat{q}'b}\left[I - \frac{\theta}{b}\right]_+. \end{aligned} \quad \text{(S67)}$$

For convenience, we denote the Gaussian variable $I = \sqrt{\alpha\hat{q}}z + \sum_{k=1}^{K}[(x^k - t^k)w^k + (y^k - s^k)v^k)]$. Each neuron receives input with mean 0, and variance (denoted σ^2) that depends on the stimuli presented – how many, and whether they are matched or mismatched. From the above equation we see that neurons' firing-rates follow a truncated Gaussian distribution. The saddle point equations [Eq. (S65)] can be simplified into,

$$\begin{aligned} \hat{q}' &= \frac{1-\mu}{1+(1-\mu)q'} + \frac{1+\mu}{1+(1+\mu)q'}, \\ \hat{q} &= \left[\left(\frac{1-\mu}{1+(1-\mu)q'}\right)^2 + \left(\frac{1+\mu}{1+(1+\mu)q'}\right)^2\right] q, \end{aligned}$$

$$
\begin{aligned}
q' &= \frac{bH\left(\frac{\theta}{b\sigma}\right)}{1+\alpha b\hat{q}'}, \\
q &= \frac{(q')^2}{H\left(\frac{\theta}{b\sigma}\right)}\left[\sigma^2 + \left(\frac{\theta}{b}\right)^2 - \frac{\sigma\theta}{\sqrt{2\pi}b}\frac{e^{-\frac{\theta^2}{2b^2\sigma^2}}}{H\left(\frac{\theta}{b\sigma}\right)}\right], \\
\delta x^k &= x^k - t^k = \frac{(1+q')x^k - \mu q' y^k}{1+2q'+(1-\mu^2)(q')^2}, \\
\delta y^k &= y^k - s^k = \frac{-\mu q' x^k + (1+q')y^k}{1+2q'+(1-\mu^2)(q')^2},
\end{aligned}
\tag{S68}
$$

where $H(x) = \int_x^\infty Dz$ is related to the complementary error function. Since $q' \geq 0$ and $x^k = y^k = 1$ in the match condition, $\delta x^k = \delta y^k \geq 0$. The variance σ^2 in the above equations is given by,

$$
\begin{aligned}
\sigma^2 &= \alpha\hat{q} + \sum_{k=1}^{K}\left[(\delta x^k)^2 + (\delta y^k)^2 + 2\mu\delta x^k \delta y^k\right] \\
&= \frac{2[(1-\mu^2)(1+q')^2+\mu^2]S + 2\mu[1-(1-\mu^2)(q')^2]T}{[1+2q'+(1-\mu^2)(q')^2]^2} \\
&+ \frac{2\alpha(q')^2}{H\left(\frac{\theta}{b\sigma}\right)}\left[\sigma^2 + \left(\frac{\theta}{b}\right)^2 - \frac{\sigma\theta}{\sqrt{2\pi}b}\frac{e^{-\frac{\theta^2}{2b^2\sigma^2}}}{H\left(\frac{\theta}{b\sigma}\right)}\right]\frac{1+\mu^2+2(1-\mu^2)q'+(1-\mu^2)+(1-\mu^2)(q')^2}{[1+2q'+(1-\mu^2)(q')^2]^2}.
\end{aligned}
\tag{S69}
$$

Here, we define variables that quantify the number of stimuli presented and whether their presentation is matched or mismatched: $S = \frac{1}{2}\sum_{k=1}^{K}[(x^k)^2 + (y^k)^2]$ and $T = \sum_{k=1}^{K} x^k y^k$. Combining Eq. (S69) with the first and third lines of Eq. (S68) gives a solution for σ, q', $\hat{q}'$. By substituting these into the other saddle point equations, we get all the order parameters.

In this case, the mean and variance of the firing-rate distribution are,

$$
\begin{aligned}
\langle r^\star \rangle &= \frac{1}{1+\alpha b\hat{q}'}\left[\frac{b\sigma}{\sqrt{2\pi}} - \theta H\left(\frac{\theta}{b\sigma}\right)\right], \\
\mathrm{Var}(r^\star) &= \frac{1}{(1+\alpha b\hat{q}')^2}\left[b^2\sigma^2\left(H\left(\frac{\theta}{b\sigma}\right) - \frac{1}{2\pi}e^{-\frac{\theta^2}{b^2\sigma^2}}\right) - \frac{\theta b\sigma}{\sqrt{2\pi}}\left(1-2H\left(\frac{\theta}{b\sigma}\right)\right)\right. \\
&\left. + \theta^2 H\left(\frac{\theta}{b\sigma}\right)\left(1-H\left(\frac{\theta}{b\sigma}\right)\right)\right].
\end{aligned}
\tag{S70}
$$

3.2.2. The case $\alpha \to 0$

When $\alpha \to 0$, the saddle point equations reduce to,

$$
\begin{aligned}
q' &= bH\left(\frac{\theta}{b\sigma}\right), \\
\sigma^2 &= \frac{2[(1-\mu^2)(1+q')^2+\mu^2]S + 2\mu[1-(1-\mu^2)(q')^2]T}{[1+2q'+(1-\mu^2)(q')^2]^2}.
\end{aligned} \tag{S71}
$$

Once the values of q' and σ are obtained from Eq. (S71), other order parameters in Eq. (S68) can be computed directly. Note that when $\theta = 0$, then $q' = b/2$. For a general threshold value $\theta \geq 0$, q' is proportional to the gain parameter b and can thus be regarded as an order parameter quantifying the 'effective gain parameter' in the network. We see from Eq. (S71) that q' depends on σ, which is scaled in turn by the quantities measuring the total stimulus strength, S and T. Thus, the changes of q' in the match versus mismatch condition can be viewed as a global gain component in the predictive signal.

The single neuron firing-rate [Eq. (S34)] is now,

$$
r^\star = \phi(bI) = [bI-\theta]_+, \qquad I = \sum_{k=1}^{K}\left[w^k \delta x^k + v^k \delta y^k\right] \sim \mathcal{N}(0,\sigma^2). \tag{S72}
$$

Notice that the mean and variance of the firing-rate [Eq. (S72)] can be obtained from Eq. (S70) by setting $\alpha = 0$, and that the variable I in this case coincides the voltage level of neurons in the network [Eq. (S67)]. These results are used to generated the firing-rate statistics in Fig. 1.

In the case where only one stimulus-pair is presented ($K = 1$), the Pearson correlation between firing-rate vectors in the mismatch and match conditions can be calculated as follows. We denote by I_x, I_y, I_{xy} the voltage levels in the x-only, y-only mismatch and match conditions, respectively. The I's are multivariate Gaussian variables with mean 0. We computed the correlations between inputs to neurons in the different mismatch conditions $\rho^I_{x,y} = (\langle I_x I_y\rangle - \langle I_x\rangle\langle I_y\rangle)/(\sigma_x\sigma_y)$ and between the mismatch and match conditions $\rho^I_{x,xy} = (\langle I_x I_{xy}\rangle - \langle I_x\rangle\langle I_{xy}\rangle)/(\sigma_x\sigma_{xy})$. Here σ_x^2, σ_y^2, σ_{xy}^2, are the variances of I_x, I_y, I_{xy}, respectively. We found,

$$
\begin{aligned}
\rho^I_{x,y} &= -\frac{2\mu[1+(1-\mu^2)(q')^2]}{(1-\mu^2)(1+q')^2+\mu^2}, \\
\rho^I_{x,xy} &= \frac{2(1+\mu)[1+(1-\mu)q']^2}{\sqrt{[(1-\mu^2)(1+q')^2+\mu^2][\mu(1+\mu)+(1-\mu^2)(1+2q'+(1-\mu)(q')^2)]}}.
\end{aligned} \tag{S73}
$$

From the symmetry in the model we have $\rho^I_{x,xy} = \rho^I_{y,xy}$.

In most cases, the experimentally accessible quantity is the firing-rate rather than the input current, so we also computed the Pearson correlation between firing-rates. We denote this correlation as $\rho^r_{m,n}$, where m, n can refer to the conditions x, y, xy, and write its formal definition,

$$\rho^r_{m,n} = \frac{\langle [bI_m - \theta]_+ [bI_n - \theta]_+ \rangle - \langle [bI_m - \theta]_+ \rangle \langle [bI_n - \theta]_+ \rangle}{\sqrt{\mathrm{Var}([bI_m - \theta]_+)\mathrm{Var}([bI_n - \theta]_+)}}. \tag{S74}$$

When $\theta = 0$, the cross covariance between firing-rates can be worked out as,

$$\langle [bI_m - \theta]_+ [bI_n - \theta]_+ \rangle = \frac{b^2 \sigma_m \sigma_n}{2\pi} \left(\frac{\pi}{2} \rho^I_{m,n} + \rho^I_{m,n} \arctan \frac{\rho^I_{m,n}}{\sqrt{1 - (\rho^I_{m,n})^2}} + \sqrt{1 - (\rho^I_{m,n})^2} \right). \tag{S75}$$

Together with the firing-rate mean and variance [Eq. (S70)], we obtained an explicit expression at for the firing-rate Pearson correlation, $\rho^r_{m,n}$. In the case of $\theta = 0$, Eq. (S74) becomes,

$$\rho^r_{m,n} = \frac{1}{\pi - 1} \left[\frac{\pi}{2} \rho^I_{m,n} + \rho^I_{m,n} \arctan \frac{\rho^I_{m,n}}{\sqrt{1 - (\rho^I_{m,n})^2}} + \sqrt{1 - (\rho^I_{m,n})^2} - 1 \right]. \tag{S76}$$

3.3. Balance level distribution

The balance level for neuron i in the network is defined as,

$$B_i = \left| \frac{I^F_i}{I^F_i - I^R_i} \right| = \left| \frac{\sum_{k=1}^P (w^k_i x^k + v^k_i y^k)}{\sum_{k=1}^P (w^k_i \delta x^k + v^k_i \delta y^k)} \right|. \tag{S77}$$

The B_i's are i.i.d. random variables for each i. Here the denominator is the net input $\delta I_i = I^F_i - I^R_i$ to neuron i, i.e., the difference between feedforward and recurrent input currents,

$$I^F = \sum_{k=1}^K (w^k x^k + v^k y^k), \qquad I^R = \sum_{k=1}^K (w^k \hat{x}^k + v^k \hat{y}^k). \tag{S78}$$

From Eq. (S66), δI can be expressed as

$$\delta I = \sum_{k=1}^P (w^k \delta x^k + v^k \delta y^k) = I - \frac{\alpha \hat{q}' b}{1 + \alpha \hat{q}' b} \left[I - \frac{\theta}{b} \right]_+, \tag{S79}$$

where $I = \sqrt{\alpha \hat{q}} z + \sum_{k=1}^{K} (w^k \delta x^k + v^k \delta y^k)$ is defined in Eq. (S67). To simplify the notation we drop the subscript i from δI. Thus, to sample from the distribution of balance levels, one can first sample (w^k, v^k, z) from their corresponding distributions and then compute I^F and δI. The ratio between I^F and δI gives a sample of the balance level.

When the synaptic weights have Gaussian distribution and $\alpha = 0$, the pair $(I^F, \delta I)$ is jointly Gaussian,

$$(I^F, \delta I) \sim \mathcal{N}\left(0, \begin{pmatrix} \sigma_F^2 & \rho_B \sigma_F \sigma_\delta \\ \rho_B \sigma_F \sigma_\delta & \sigma_\delta^2 \end{pmatrix}\right). \tag{S80}$$

The coefficients of the covariance matrix of $(I^F, \delta I)$ are,

$$\begin{aligned} \sigma_F^2 &= 2\left(S + \mu T\right), \\ \sigma_\delta^2 &= \frac{2[(1-\mu^2)(1+q')^2 + \mu^2]S + 2\mu[1-(1-\mu^2)(q')^2]T}{[1+2q'+(1-\mu^2)(q')^2]^2}, \\ \rho_B \sigma_F \sigma_\delta &= \frac{2[1+(1-\mu^2)q']S + 2\mu T}{1+2q'+(1-\mu^2)(q')^2}. \end{aligned} \tag{S81}$$

The balance level in this case can be expressed using a Cauchy random variable ξ as,

$$B = \frac{\sigma_F}{\sigma_\delta} |\xi|, \tag{S82}$$

where the probability density function for $\xi \in \mathbb{R}$ is,

$$p(\xi) = \frac{1}{\pi} \frac{\sqrt{1-\rho_B^2}}{(\xi - \rho_B)^2 + 1 - \rho_B^2}. \tag{S83}$$

This result means that the average of the balance level distribution diverges. We use the quantiles to measure the magnitude of the balance level in the network (Fig. 2).

4. CHARACTERIZING DIFFERENT FUNCTIONAL NEURON TYPES

4.1. Firing-rate correlations from two-body replica calculations

In this section we compute the probabilities of single neurons belonging to the different functional cell types for two stimulus-pairs. Since the stimulus-pairs and the neurons are statistically equivalent, we focus on the responses of neuron i to the first two stimulus-pairs, $(h_i^{x_1}, h_i^{y_1}, h_i^{x_1 y_1}, h_i^{x_2}, h_i^{y_2}, h_i^{x_2 y_2})$. To mathematically characterize those voltage responses, we consider the joint distribution of the neurons' firing-rates in two different stimulus con-

ditions,

$$p(r_1, r_2) = \frac{1}{N} \sum_{i=1}^{N} \delta(r_1 - r_i^A)\delta(r_2 - r_i^B). \tag{S84}$$

The superscripts A, B denote the stimulus conditions, i.e., A and B are chosen from $\{x_1, y_1, x_1y_1, x_2, y_2, x_2y_2\}$. We will show that at the limit $N \to \infty$, the joint distribution for all different combinations of stimulus conditions can be obtained from the calculation of pairwise firing-rate correlations [Eq. (S84)].

To evaluate Eq. (S84), we consider two identical networks driven by different stimulus inputs. The energy function of the 1st system with firing-rates $\boldsymbol{r}^A$ is,

$$E_0^A(\boldsymbol{r}^A; \{\boldsymbol{w}^k, \boldsymbol{v}^k\}) = \sum_{k=1}^{P} b \left(-x_A^k \boldsymbol{w}^k \cdot \boldsymbol{r}^A - y_A^k \boldsymbol{v}^k \cdot \boldsymbol{r}^A + \frac{1}{2N} \left[(\boldsymbol{w}^k \cdot \boldsymbol{r}^A)^2 + (\boldsymbol{v}^k \cdot \boldsymbol{r}^A)^2 \right] \right) + \sum_{i=1}^{N} F(r_i^A), \tag{S85}$$

and similarly for the energy function of the 2nd system, $E_0^B(\boldsymbol{r}^B; \{\boldsymbol{w}^k, \boldsymbol{v}^k\})$. Note that in stimulus conditions A and B, only the first two stimulus-pair inputs are nonzero.

The partition function of the whole system is defined as

$$Z_{\text{total}} = \int_{\mathbb{R}_+^{2N}} e^{-\beta E_0^A(\boldsymbol{r}^A; \{\boldsymbol{w}^k, \boldsymbol{v}^k\}) - \beta E_0^B(\boldsymbol{r}^B; \{\boldsymbol{w}^k, \boldsymbol{v}^k\})} \mathrm{d}\boldsymbol{r}^A \mathrm{d}\boldsymbol{r}^B = Z_A \cdot Z_B. \tag{S86}$$

Again we use the replica trick,

$$\lim_{N\to\infty} \frac{\ln Z_{\text{total}}}{2N} = \lim_{N\to\infty} \left\langle \frac{\ln Z_{\text{total}}}{2N} \right\rangle_{\boldsymbol{w},\boldsymbol{v}} = \lim_{n\to0} \lim_{N\to\infty} \frac{\ln \langle Z_{\text{total}}^n \rangle}{2nN} = \lim_{n\to0} \lim_{N\to\infty} \frac{\ln \langle Z_A^n Z_B^n \rangle}{2nN}. \tag{S87}$$

Note that the neural activities $\boldsymbol{r}^A$ and $\boldsymbol{r}^B$ of the two separate but identical networks are in fact statistically coupled due to the replica-average over $(\boldsymbol{w}^k, \boldsymbol{v}^k)$. The calculation for $\langle Z_A^n Z_B^n \rangle$ is similar to the one shown in §3. We denote the order parameters under replica symmetric ansatz as,

$$\begin{aligned} q_A^{ab} =& \frac{1}{N} \sum_j r_j^{A,a} r_j^{A,b} = q_0^A \delta_{ab} + q_1^A (1 - \delta_{ab}), \\ q_B^{ab} =& \frac{1}{N} \sum_j r_j^{B,a} r_j^{B,b} = q_0^B \delta_{ab} + q_1^B (1 - \delta_{ab}), \\ q_c^{ab} =& \frac{1}{N} \sum_j r_j^{A,a} r_j^{B,b} = q_{c,0} \delta_{ab} + q_{c,1} (1 - \delta_{ab}). \end{aligned} \tag{S88}$$

The last order parameter represents the overlap between replicas in system A and system

B. Thus, the calculation of firing-rate correlations is very similar to the one-step replica symmetry-breaking calculation where the overlap between replicas within the same system is different from the overlap between the systems [9].

In the $N, P \to \infty, P/N \to \alpha, n \to 0$ limit, with similar changes of variables as before [Eq. (S58)], we write the result of the calculation as,

$$\frac{\ln \langle Z_A^n Z_B^n \rangle}{nN} = \mathcal{F}_{\text{total}}(q, \hat{q}, q', \hat{q}', t, s). \tag{S89}$$

Each order parameter in the function $\mathcal{F}_{\text{total}}$ has 3 components. For example, q has the components (q_A, q_B, q_c). The calculation gives the function $\mathcal{F}_{\text{total}}$,

$$\begin{aligned}
&\mathcal{F}_{\text{total}}(q, \hat{q}, q', \hat{q}', t, s) = \\
&\quad \frac{g\beta}{2} \sum_k \left((t_A^k)^2 + (s_A^k)^2 + (t_B^k)^2 + (s_B^k)^2 \right) + \frac{\alpha}{2} \left(\hat{q}'_A q'_A + \hat{q}'_B q'_B + \hat{q}'_c q'_c \right) \\
&\quad + \frac{\alpha g \beta}{2} \left(\hat{q}_A q'_A - \hat{q}_A q'_A + \hat{q}'_B q_B - \hat{q}_B q'_B + \hat{q}'_c q_c - \hat{q}_c q'_c \right) \\
&\quad + \int D\boldsymbol{z} \ln \left[\int \mathrm{d}\nu_\beta(r_A) \mathrm{d}\nu_\beta(r_B) \left\langle e^{-\beta \mathcal{G}(r_A, r_B, \boldsymbol{z}, w^k, v^k)} \right\rangle \right] - \lim_{n \to 0} \frac{1}{2n} \left\langle \ln \det \mathcal{A}(\mu, q) \right\rangle_\mu .
\end{aligned} \tag{S90}$$

We introduced the functions,

$$\begin{aligned}
&\mathcal{G}(r_A, r_B, \boldsymbol{z}, w^k, v^k) = \\
&\quad \frac{g\alpha}{2} \hat{q}'_A r_A^2 - g r_A \sqrt{\alpha} \left(\sqrt{\hat{q}_c} z_1 + \sqrt{\hat{q}_A - \hat{q}_c} z_2 \right) - g r_A \sum_k \left[(x_A^k - t_A^k) w^k + (y_A^k - s_A^k) v^k \right] \\
&\quad + \frac{g\alpha}{2} \hat{q}'_B r_B^2 - g r_B \sqrt{\alpha} \left(\sqrt{\hat{q}_c} z_1 + \sqrt{\hat{q}_B - \hat{q}_c} z_3 \right) - g r_B \sum_k \left[(x_B^k - t_B^k) w^k + (y_B^k - s_B^k) v^k \right] \\
&\quad - g \alpha \hat{q}'_c r_A r_B, \\
&\mathcal{A}(\mu, q) = \begin{pmatrix} \mathcal{A}_{11} & \mathcal{A}_{12} \\ \mathcal{A}_{12} & \mathcal{A}_{22} \end{pmatrix} \\
&\qquad \mathcal{A}_{11} = \begin{pmatrix} (1 + q'_A) I_n + g\beta q_A \mathbf{1}\mathbf{1}^\top & \mu(q'_A I_n + g\beta q_A \mathbf{1}\mathbf{1}^\top) \\ \mu(q'_A I_n + g\beta q_A \mathbf{1}\mathbf{1}^\top) & (1 + q'_A) I_n + g\beta q_A \mathbf{1}\mathbf{1}^\top \end{pmatrix} \\
&\qquad \mathcal{A}_{12} = \begin{pmatrix} q'_c I_n + g\beta q_c \mathbf{1}\mathbf{1}^\top & \mu(q'_c I_n + g\beta q_c \mathbf{1}\mathbf{1}^\top) \\ \mu(q'_c I_n + g\beta q_c \mathbf{1}\mathbf{1}^\top) & q'_c I_n + g\beta q_c \mathbf{1}\mathbf{1}^\top \end{pmatrix}
\end{aligned} \tag{S91}$$

From symmetry, $\mathcal{A}_{22}$ is obtained by replacing $A \leftrightarrow B$ in $\mathcal{A}_{11}$. Here, I_n is the $n \times n$ identity matrix and $\mathbf{1}$ is an n-dimensional vector of 1's. All the order parameters in Eq. (S90) should be evaluated at the saddle point, in the limit $\beta \to \infty$. We obtain these order parameters as

follows.

First, we find the Hamiltonian corresponding to this system,

$$
\begin{aligned}
\mathcal{H}(r_A, r_B) &= \mathcal{G}(r_A, r_B, \boldsymbol{z}, w^k, v^k) + F(r_A) + F(r_B) \\
&= \frac{g\alpha}{2}\hat{q}'_A r_A^2 - g r_A \sqrt{\alpha}\left(\sqrt{\hat{q}_c} z_1 + \sqrt{\hat{q}_A - \hat{q}_c} z_2\right) - g r_A \sum_k \left[(x_A^k - t_A^k) w^k + (y_A^k - s_A^k) v^k\right] \\
&+ \frac{g\alpha}{2}\hat{q}'_B r_B^2 - g r_B \sqrt{\alpha}\left(\sqrt{\hat{q}_c} z_1 + \sqrt{\hat{q}_B - \hat{q}_c} z_3\right) - g r_B \sum_k \left[(x_B^k - t_B^k) w^k + (y_B^k - s_B^k) v^k\right] \\
&- g\alpha \hat{q}'_c r_A r_B + F(r_A) + F(r_B). \qquad \text{(S92)}
\end{aligned}
$$

The extra terms $F(r_A)$ and $F(r_B)$ come from the probability measure $\mathrm{d}\nu_\beta$. When $\beta \to \infty$, the unique minimum $(r_A^\star, r_B^\star)$ is given by,

$$
\begin{aligned}
r_A^\star &= \phi\left(-g\alpha\hat{q}'_A r_A^\star + g\sqrt{\alpha}\left(\sqrt{\hat{q}_c} z_1 + \sqrt{\hat{q}_A - \hat{q}_c} z_2\right) + g\sum_{k=1}^K \left[(x_A^k - t_A^k) w^k + (y_A^k - s_A^k) v^k)\right] + g\alpha\hat{q}'_c r_B^\star\right), \\
r_B^\star &= \phi\left(-g\alpha\hat{q}'_B r_B^\star + g\sqrt{\alpha}\left(\sqrt{\hat{q}_c} z_1 + \sqrt{\hat{q}_B - \hat{q}_c} z_3\right) + g\sum_{k=1}^K \left[(x_B^k - t_B^k) w^k + (y_B^k - s_B^k) v^k)\right] + g\alpha\hat{q}'_c r_A^\star\right).
\end{aligned}
\qquad \text{(S93)}
$$

At the saddle point, the derivative of $\mathcal{F}_{\text{total}}$ [Eq. (S90)] with respect to $\hat{q}_c$ is set to 0, giving

$$
0 = \frac{\alpha g \beta}{2}\left\{-q'_c + \frac{1}{\sqrt{\alpha}}\int \mathrm{d}Q_\beta \left[\left(\frac{z_1}{\sqrt{\hat{q}_c}} - \frac{z_2}{\sqrt{\hat{q}_A - \hat{q}_c}}\right) r_A + \left(\frac{z_1}{\sqrt{\hat{q}_c}} - \frac{z_3}{\sqrt{\hat{q}_B - \hat{q}_c}}\right) r_B\right]\right\}. \qquad \text{(S94)}
$$

In the limit $\beta \to \infty$ we find that,

$$
\left\langle \left(\frac{z_1}{\sqrt{\hat{q}_c}} - \frac{z_2}{\sqrt{\hat{q}_A - \hat{q}_c}}\right) r_A^\star \right\rangle_z = \left\langle \frac{1}{\sqrt{\hat{q}_c}}\frac{\partial r_A^\star}{\partial z_1} - \frac{1}{\sqrt{\hat{q}_A - \hat{q}_c}}\frac{\partial r_A^\star}{\partial z_2} \right\rangle_z \overset{\text{Eq. (S93)}}{=} 0. \qquad \text{(S95)}
$$

Similarly, the average over the term proportional to $r_B^\star$ in Eq. (S94) is also 0. Substituting this into Eq. (S94), we get at the saddle point,

$$
q'_c = 0. \qquad \text{(S96)}
$$

Next, we simplify the determinant of $\mathcal{A}(\mu, q)$. It is useful to write the submatrices as,

$$
\begin{aligned}
\mathcal{A}_{11} &= I_{2n} + q'_A \begin{pmatrix} 1 & \mu \\ \mu & 1 \end{pmatrix} \otimes I_n + g\beta q_A \begin{pmatrix} 1 & \mu \\ \mu & 1 \end{pmatrix} \otimes \mathbf{1}\mathbf{1}^\top, \\
\mathcal{A}_{12} &= q'_c \begin{pmatrix} 1 & \mu \\ \mu & 1 \end{pmatrix} \otimes I_n + g\beta q_c \begin{pmatrix} 1 & \mu \\ \mu & 1 \end{pmatrix} \otimes \mathbf{1}\mathbf{1}^\top. \qquad \text{(S97)}
\end{aligned}
$$

The symbol $\otimes$ denotes the Kronecker product between two matrices. For this product, we have the identity, $(A\otimes B)(C\otimes D)=(AC)\otimes(BD)$. Therefore the submatrices $\mathcal{A}_{11}$ and $\mathcal{A}_{12}$ commute and the determinant of $\mathcal{A}(\mu,q)$ becomes,

$$\det\mathcal{A}(\mu,q)=\det\begin{pmatrix}\mathcal{A}_{11} & \mathcal{A}_{12}\\ \mathcal{A}_{12} & \mathcal{A}_{22}\end{pmatrix}=\det(\mathcal{A}_{11}\mathcal{A}_{22}-\mathcal{A}_{12}^2). \tag{S98}$$

The two terms are equal to,

$$\begin{aligned}\mathcal{A}_{11}\mathcal{A}_{22}&=I_{2n}+(q_A'+q_B')\begin{pmatrix}1 & \mu\\ \mu & 1\end{pmatrix}\otimes I_n+q_A'q_B'\begin{pmatrix}1+\mu^2 & 2\mu\\ 2\mu & 1+\mu^2\end{pmatrix}\otimes I_n\\ &\quad+g\beta(ng\beta q_Aq_B+q_Aq_B'+q_A'q_B)\begin{pmatrix}1+\mu^2 & 2\mu\\ 2\mu & 1+\mu^2\end{pmatrix}\otimes\mathbf{1}\mathbf{1}^\top,\\ \mathcal{A}_{12}^2&=(2g\beta q_c'q_c+ng^2\beta^2q_c^2+O(q_c'^2))\begin{pmatrix}1+\mu^2 & 2\mu\\ 2\mu & 1+\mu^2\end{pmatrix}\otimes\mathbf{1}\mathbf{1}^\top,\end{aligned} \tag{S99}$$

where we used $q_c'=0$. Note that we have kept the term linear in q_c' in $\mathcal{A}_{12}^2$, anticipating that we will need to evaluate derivatives with respect to q_c' below. We find that the determinant has the following form,

$$\begin{aligned}\frac{1}{n}\ln\det(\mathcal{A}_{11}\mathcal{A}_{22}-\mathcal{A}_{12}^2)&=\frac{1}{n}\ln\det(Q_0\otimes I_n+Q_1(n)\otimes\mathbf{1}\mathbf{1}^\top)\\ &=\ln\det Q_0+\frac{1}{n}\ln\frac{\det(Q_0+nQ_1(n))}{\det Q_0}\\ &\overset{n\to0}{=}\ln\det Q_0+\mathrm{tr}[Q_0^{-1}Q_1(0)].\end{aligned} \tag{S100}$$

Here, Q_0 and $Q_1(n)$ are 2×2 matrices that depend on the order parameters,

$$\begin{aligned}Q_0&=\begin{pmatrix}1+q_A' & \mu q_A'\\ \mu q_A' & 1+q_A'\end{pmatrix}\begin{pmatrix}1+q_B' & \mu q_B'\\ \mu q_B' & 1+q_B'\end{pmatrix},\\ Q_1(n)&=\left[g\beta(q_A'q_B+q_Aq_B')+ng^2\beta^2(q_Aq_B-q_c^2)-2g\beta q_c'q_c+O(q_c'^2)\right]\begin{pmatrix}1+\mu^2 & 2\mu\\ 2\mu & 1+\mu^2\end{pmatrix}.\end{aligned} \tag{S101}$$

We use the above simplification to evaluate the derivative of $\mathcal{F}_{\text{total}}$ [Eq. (S90)] with respect to q_c and set it to 0 at the saddle point,

$$0=\frac{\alpha g\beta}{2}\left[\hat{q}_c'-\frac{1}{2g\beta}\left\langle\mathrm{tr}\left[Q_0^{-1}\frac{\partial Q_1(0)}{\partial q_c}\right]\right\rangle_\mu\right]. \tag{S102}$$

Using Eq. (S101), we find that $\frac{\partial Q_1(0)}{\partial q_c}|_{q_c'=0} = 0$. Therefore,

$$\hat{q}_c' = 0. \tag{S103}$$

Substituting this result into Eq. (S93), we see this is consistent with the one-body replica results in Eq. (S34). Moreover, all the saddle point equations in the one-body scenario [Eq. (S60)] will hold in the two-body scenario. The two new equations when taking derivatives of $\mathcal{F}_{\text{total}}$ with respect to $\hat{q}_c'$ and q_c' are,

$$\begin{aligned} 0 &= \frac{\partial \mathcal{F}_{\text{total}}}{\partial \hat{q}_c'} = \frac{\alpha g \beta}{2} \left(\frac{q_c'}{g\beta} + q_c - \int \mathrm{d}Q_\beta r_A r_B \right), \\ 0 &= \frac{\partial \mathcal{F}_{\text{total}}}{\partial q_c'} = \frac{\alpha g \beta}{2} \left[-\frac{\hat{q}_c'}{g\beta} - \hat{q}_c - \frac{1}{2g\beta} \left\langle \operatorname{tr} \left[Q_0^{-1} \frac{\partial Q_1(0)}{\partial q_c'} \right] \right\rangle_\mu \right]. \end{aligned} \tag{S104}$$

The second equation can be further simplified as follows. From Eq. (S101), we find

$$\left. \frac{\partial Q_1(0)}{\partial q_c'} \right|_{q_c'=0} = -2 q_c g \beta \begin{pmatrix} 1+\mu^2 & 2\mu \\ 2\mu & 1+\mu^2 \end{pmatrix}. \tag{S105}$$

Combining with Eq. (S101), we get

$$\begin{aligned} \frac{1}{2g\beta} \operatorname{tr} \left[Q_0^{-1} \frac{\partial Q_1(0)}{\partial q_c'} \right] \Bigg|_{q_c'=0} &= - \frac{(1-\mu)^2 q_c}{[1+(1-\mu)q_A'][1+(1-\mu)q_B']} - \frac{(1+\mu)^2 q_c}{[1+(1+\mu)q_A'][1+(1+\mu)q_B']} \\ &\equiv -C(\mu, q_A', q_B') q_c. \end{aligned} \tag{S106}$$

Therefore, using Eqs. (S104-S106), in the limit $\beta \to \infty$, we get,

$$\hat{q}_c = \langle C(\mu, q_A', q_B') \rangle_\mu \, q_c = \langle C(\mu, q_A', q_B') \rangle_\mu \, \langle r_A^\star r_B^\star \rangle_{w^k, v^k, \boldsymbol{z}}. \tag{S107}$$

Below we consider the case where the activation function ϕ is ReLU and μ's are the same for all learned stimulus-pairs. In this case $r_A^\star$ and $r_B^\star$ can be solved in closed form,

$$\begin{aligned} r_A^\star(w^k, v^k, \boldsymbol{z}) &= \frac{g}{1+\alpha \hat{q}_A' g} \left[I_A - \frac{\theta}{g} \right]_+, \\ r_B^\star(w^k, v^k, \boldsymbol{z}) &= \frac{g}{1+\alpha \hat{q}_B' g} \left[I_B - \frac{\theta}{g} \right]_+, \\ \frac{\hat{q}_c}{C(\mu, q_A', q_B')} &= q_c = \frac{g^2}{(1+\alpha g \hat{q}_A')(1+\alpha g \hat{q}_B')} \left\langle \left[I_A - \frac{\theta}{g} \right]_+ \left[I_B - \frac{\theta}{g} \right]_+ \right\rangle_{I_A, I_B}. \end{aligned} \tag{S108}$$

The random variables representing the currents can be read from Eq. (S93),

$$I_A = \sqrt{\alpha}\left(\sqrt{\hat{q}_c}z_1 + \sqrt{\hat{q}_A - \hat{q}_c}z_2\right) + \sum_{k=1}^{K}\left[(x_A^k - t_A^k)w^k + (y_A^k - s_A^k)v^k)\right],$$
$$I_B = \sqrt{\alpha}\left(\sqrt{\hat{q}_c}z_1 + \sqrt{\hat{q}_B - \hat{q}_c}z_3\right) + \sum_{k=1}^{K}\left[(x_B^k - t_B^k)w^k + (y_B^k - s_B^k)v^k)\right]. \quad \text{(S109)}$$

In summary, to obtain the joint distribution of neural activity under two stimulus conditions A and B, we first sample w^k, v^k, $\boldsymbol{z}$ from their corresponding distributions, and calculate I_A and I_B from Eq. (S109). The order parameters $q_A, q'_A, q_B, q'_B, \hat{q}_A, \hat{q}'_A, \hat{q}_B, \hat{q}'_B$ are then solved from the one-body replica equations [Eq. (S60)] and order parameters introduced in the two-body calculation $(\hat{q}_c, \hat{q}'_c, q_c, q'_c)$ are obtained from Eq. (S108). Finally, $r^\star_A$ and $r^\star_B$ are calculated from Eq. (S93), which gives a random sample from the joint distribution.

The joint distribution of voltage levels can be computed similarly using the following formula for $h^\star_A$,

$$h^\star_A(w^k, v^k, \boldsymbol{z}) = I_A - \frac{\alpha\hat{q}'_A g}{1+\alpha\hat{q}'_A g}\left[I_A - \frac{\theta}{g}\right]_+. \quad \text{(S110)}$$

A similar formula holds for $h^\star_B$ with the corresponding input current and order parameters. These results can be generalized to scenarios with more than two stimulus conditions. This joint distribution was be used to calculate the fraction of different functional neuronal types as shown in Fig. 3b,d.

4.2. Explicit formulas in the Gaussian case

When the weights w^k and v^k are Gaussian, the input currents I_A and I_B are also Gaussian variables. Moreover, their variances $\sigma_A^2 = \langle I_A^2\rangle$ and $\sigma_B^2 = \langle I_B^2\rangle$ are given by the one-body calculation [Eq. (S69)]. We denote the input current covariance as $\sigma_{AB} = \langle I_A I_B\rangle$. This covariance is given by,

$$\sigma_{AB} = \alpha\hat{q}_c + \sum_{k=1}^{K}\left[\delta x_A^k \delta x_B^k + \mu^k(\delta x_A^k \delta y_B^k + \delta x_B^k \delta y_A^k) + \delta y_A^k \delta y_B^k\right] \equiv \alpha\hat{q}_c + \sigma^0_{AB}. \quad \text{(S111)}$$

Here σ^0_{AB} can be obtained from the one-body replica equations [Eq. (S60)].

Substituting σ_{AB} this into Eq. (S108) (i.e., averaging over the correlated Gaussians I_A, I_B), and defining $\rho_{AB} = \sigma_{AB}/(\sigma_A\sigma_B)$, we get a self-consistent equation for ρ_{AB},

$$\rho_{AB} - \frac{\sigma^0_{AB}}{\sigma_A\sigma_B} = \frac{\alpha b^2 C(\mu, q'_A, q'_B)\sqrt{1-\rho^2_{AB}}}{(1+\alpha b\hat{q}'_A)(1+\alpha b\hat{q}'_B)}$$

$$\times \int_{\frac{\theta}{b}}^{+\infty} Dz \left[\frac{1}{2\pi} e^{-\frac{(b\rho_{AB}z-\theta)^2}{2b^2(1-\rho_{AB}^2)}} + \frac{b\rho_{AB}z-\theta}{b\sqrt{2\pi(1-\rho_{AB}^2)}} H\left(-\frac{b\rho_{AB}z-\theta}{b\sqrt{1-\rho_{AB}^2}}\right)\right]\left(z-\frac{\theta}{b}\right). \tag{S112}$$

When $\theta = 0$, the above equation for ρ_{AB} simplifies into

$$\rho_{AB} - \frac{\sigma_{AB}^0}{\sigma_A \sigma_B} = \frac{\alpha b^2 C(\mu, q'_A, q'_B)}{2\pi(1+\alpha b\hat{q}'_A)(1+\alpha b\hat{q}'_B)} \left(\frac{\pi}{2}\rho_{AB} + \rho_{AB} \arctan\frac{\rho_{AB}}{\sqrt{1-\rho_{AB}^2}} + \sqrt{1-\rho_{AB}^2}\right). \tag{S113}$$

Note that the quantity ρ_{AB} calculated here is the high-dimensional counterpart of Eq. (S73), i.e., ρ_{AB} reduces to $\rho_{m,n}^I$ in the limit $\alpha \to 0$. We computed the firing rate correlations in the high-dimensional regime based on Eqs. (S74-S76) which give the correlation between the input current ρ_{AB}.

The fraction of different functional neuronal types can also be obtained from the statistics σ_A^2, σ_B^2 and ρ_{AB}. Specifically, we set the stimulus conditions $A =$'x-only mismatch condition' and $B =$ 'match condition'. The fraction of PE and R neurons are defined as (Methods),

$$\begin{aligned} f_{PE} &= \mathbb{P}\left\{h_A > \frac{\sigma}{2}, h_A - h_B > \frac{\sigma}{2}\right\}, \\ f_R &= \mathbb{P}\left\{h_A > \frac{\sigma}{2}, |h_A - h_B| < \frac{\sigma}{2}\right\}, \end{aligned} \tag{S114}$$

where h_A, h_B are given by Eq. (S110). In the low-dimensional limit $\alpha \to 0$, $h_A = I_A, h_B = I_B$ and have multivariate Gaussian distribution. There the fractions of PE and R neurons have the explicit formulas,

$$\begin{aligned} f_{PE} &= \int_{\frac{\sigma}{2\sigma_A}}^{+\infty} Dz\, H\left(\frac{\frac{\sigma}{2\sigma_A} - (\frac{\sigma_A}{\sigma_B} - \rho_{AB})z}{\sqrt{1-(\frac{\sigma_A}{\sigma_B} - \rho_{AB})^2}}\right), \\ f_R &= \int_{\frac{\sigma}{2\sigma_A}}^{+\infty} Dz \left[1 - H\left(\frac{\frac{\sigma}{2\sigma_A} - (\frac{\sigma_A}{\sigma_B} - \rho_{AB})z}{\sqrt{1-(\frac{\sigma_A}{\sigma_B} - \rho_{AB})^2}}\right)\right]. \end{aligned} \tag{S115}$$

4.3. Imperfect match of paired stimuli

We consider a network that learns a single stimulus association, and is presented with a 'probe' stimulus that is an imperfect match to the expected (learned) stimulus. This difference is modeled by letting the recurrent weight vector $\boldsymbol{w}$ be different from the feedforward

weight vector $\boldsymbol{w}'$, giving the dynamics,

$$\frac{\mathrm{d}h_i(t)}{\mathrm{d}t} = -h_i(t) - \frac{b}{N}\sum_{j=1}^{N}(w_i w_j + v_i v_j)\phi(h_j(t)) + b\,(w_i' x + v_i y). \tag{S116}$$

We used this model to understand recent experimental findings, where a motor-auditory association was learned, and animals were probed with sounds that differed from the learned tone [11]. We assume that the components of $\boldsymbol{w}'$ have mean 0 and unit variance [similarly to $\boldsymbol{w}$ and $\boldsymbol{v}$, Eq. (S9)], and the following cross terms,

$$\langle w_i w_j' \rangle = \delta_{ij}\kappa, \qquad \langle v_i w_j' \rangle = \delta_{ij}\kappa\mu. \tag{S117}$$

Here $0 \leq \kappa \leq 1$ indicates the similarity between the learned stimulus input x and the one used as a probe. When $\kappa = 1$, the learned and probe stimuli are equal.

This network is very similar to the special case $\alpha \to 0$ of the network studied in §3.2.2. To understand its steady-state response, we use Eq. (S34) and define similarly,

$$r^{\kappa} = \phi(I^{\kappa}) = [bI^{\kappa} - \theta]_{+}, \qquad I^{\kappa} = w'x - w\hat{x} + v(y - \hat{y}). \tag{S118}$$

Here ϕ is assumed to be the ReLU function, $\hat{x}$ and $\hat{y}$ are the internal predictions [Eq. (S1)] and are given by the saddle point equations [Eq. (S68)],

$$\begin{aligned}\hat{x} &= \frac{\kappa[q' + (1-\mu^2)(q')^2]x + \mu q' y}{1 + 2q' + (1-\mu^2)(q')^2},\\ \hat{y} &= \frac{\kappa\mu q' x + [q' + (1-\mu^2)(q')^2]y}{1 + 2q' + (1-\mu^2)(q')^2}.\end{aligned} \tag{S119}$$

Note that we have modified them accordingly to account for fact that stimulus-pairing is 'imperfect'. When all the weights have Gaussian distributions, the order parameters q', σ satisfy [similarly to Eq. (S71)],

$$\begin{aligned} q' &= bH\left(\frac{\theta}{b\sigma^{\kappa}}\right),\\ (\sigma^{\kappa})^2 &= 2(1-\kappa^2) + \frac{2\kappa^2[(1-\mu^2)(1+q')^2 + \mu^2]S + 2\kappa\mu[1 - (1-\mu^2)(q')^2]T}{[1 + 2q' + (1-\mu^2)(q')^2]^2}.\end{aligned} \tag{S120}$$

We computed the representation similarity between stimuli semi-analytically by first solving q', σ^{κ}, sampling I^{κ} from $\mathcal{N}(0, (\sigma^{\kappa})^2)$, and finally calculating the Pearson correlation coefficient [Eq. (S74)] between $r^{\kappa=1}$ and r^{κ} for different values of κ.

To get the segregation index, we considered the difference between mismatch and match

responses Δ for an arbitrary κ and $\kappa = 1$,

$$\Delta^{\kappa} = [bI_x^{\kappa} - \theta]_+ - [bI_{xy}^{\kappa} - \theta]_+,$$
$$\Delta^{\kappa=1} = [bI_x^{\kappa=1} - \theta]_+ - [bI_{xy}^{\kappa=1} - \theta]_+. \tag{S121}$$

Note that I_x^{κ}, I_{xy}^{κ}, $I_x^{\kappa=1}$ and $I_{xy}^{\kappa=1}$ are random variables that depend on the random weights w', w and v, order parameters $\hat{x}$ and $\hat{y}$, and the inputs x and y. The inputs were chosen according to the stimulus condition (match/mismatch). The segregation index (as a function of κ) is defined as the Pearson correlation between the two random variables Δ^{κ} and $\Delta^{\kappa=1}$, which is shown in Fig. 4f.

5. THE E/I NETWORK MODEL

5.1. Derivation of the E/I connectivity in the model

We consider a network with two separate populations of excitatory and inhibitory neurons. The time-dependent voltages of E and I neurons are given by the following system of differential equations,

$$\tau_E \frac{\mathrm{d}h_i^E}{\mathrm{d}t} = -h_i^E + \sum_{j=1}^{N_E} J_{ij}^{EE} \phi(h_j^E) - \sum_{j=1}^{N_I} J_{ij}^{EI} \phi_I(h_j^I) + I_i^E,$$
$$\tau_I \frac{\mathrm{d}h_i^I}{\mathrm{d}t} = -h_i^I + \sum_{j=1}^{N_E} J_{ij}^{IE} \phi(h_j^E) - \sum_{j=1}^{N_I} J_{ij}^{II} \phi_I(h_j^I) + I_i^I. \tag{S122}$$

We assume that the activation function of inhibitory neurons is ReLU with threshold value equal to zero, $\phi_I(x) = \max\{x, 0\}$. Notice the negative sign of the third term in both equations. This implies that the connectivity matrices J^{EE}, J^{EI}, J^{IE} and J^{II} are non-negative. We now derive these matrices, and the inputs I^E and I^I, by matching the steady state activity of E neurons in the E/I network to the neural activity in the original network [Eq. (S4)]. At steady state, Eq. (S122) reads,

$$h_i^E = \sum_{j=1}^{N_E} J_{ij}^{EE} \phi(h_j^E) - \sum_{j=1}^{N_I} J_{ij}^{EI} \phi_I(h_j^I) + I_i^E,$$
$$h_i^I = \sum_{j=1}^{N_E} J_{ij}^{IE} \phi(h_j^E) - \sum_{j=1}^{N_I} J_{ij}^{II} \phi_I(h_j^I) + I_i^I. \tag{S123}$$

We restrict ourselves to choices of connectivity in which inhibitory neurons operate in the linear regime, i.e., $h_i^I \geq 0 \Rightarrow \phi_I(h_i^I) = h_i^I$. Substituting h_i^I into h_i^E in Eq. (S123) we get,

$$h_i^E = \sum_{j=1}^{N_E} \left[J_{ij}^{EE} - (J^{IE}(I_{N_I} + J^{II})^{-1} J^{EI})_{ij} \right] \phi(h_j^E) + I_i^E - \sum_{j=1}^{N_I} J_{ij}^{EI} I_j^I. \tag{S124}$$

One can be check that the steady state solution is stable when $\tau_I \ll \tau_E$. Here $(I_{N_I} + J^{II})$ is assumed to be invertible. From now on we suppress the subscript N_I indicating the dimension of the identity matrix I_{N_I}. Equating this with the steady state in the original network [Eq. (S8)] gives the constraints on the connectivity and input,

$$\begin{aligned} J_{ij}^{EE} - [J^{EI}(I + J^{II})^{-1} J^{IE}]_{ij} &= -\frac{b}{N} \sum_{k=1}^{P} \left(w_i^k w_j^k + v_i^k v_j^k \right), \\ I_i^E - [J^{EI}(I + J^{II})^{-1} I^I]_i &= b \sum_{k=1}^{P} (w_i^k x^k + v_i^k y^k). \end{aligned} \tag{S125}$$

Following a scheme for separating E/I connectivity used in previous work [12], we define positive random variables $\xi_i^k, \eta_i^k \geq 0$ such that the variables w_i^k, v_i^k are retrieved when the mean is subtracted from the new variables. Mathematically,

$$w_i^k = \xi_i^k - \bar{\xi}, \qquad v_i^k = \eta_i^k - \bar{\eta}. \tag{S126}$$

The means $\bar{\xi}$, $\bar{\eta}$ are chosen to be independent of the neuron and pattern indices i, k. Using the same trick as Ref. [12], the first equation in Eq. (S125) can be separated into two parts,

$$\begin{aligned} J_{ij}^{EE} &= \frac{\gamma b}{N} \sum_{k=1}^{P} \left(\xi_i^k \xi_j^k + \eta_i^k \eta_j^k \right) \\ &\quad + \frac{bP}{N} \left[\left(\sum_{k=1}^{P} \xi_i^k \right) \left(\sum_{k=1}^{P} \xi_j^k \right) + \left(\sum_{k=1}^{P} \eta_i^k \right) \left(\sum_{k=1}^{P} \eta_j^k \right) \right] \\ [J^{EI}(I + J^{II})^{-1} J^{IE}]_{ij} &= \frac{(\gamma + 1)b}{N} \sum_{k=1}^{P} \left(\xi_i^k \xi_j^k + \eta_i^k \eta_j^k \right) \end{aligned} \tag{S127}$$

Here γ is an arbitrary positive number, which we set to 1 in all later results.

We make two additional assumptions: (i) 'Feedforward' stimulus input exclusively target excitatory neurons ($I_i^I = 0$); and (ii) I-to-E connectivity has the form $J^{EI} = \tilde{J}^{EI}(I + J^{II})$,

where $\tilde{J}^{EI}$ is a nonnegative matrix. Given these, Eqs. (S125, S127) become,

$$
\begin{aligned}
[\tilde{J}^{EI} J^{IE}]_{ij} &= \frac{2b}{N} \sum_{k=1}^{P} \left(\xi_i^k \xi_j^k + \eta_i^k \eta_j^k \right), \\
I_i^E &= b \sum_{k=1}^{P} (w_i^k x^k + v_i^k y^k).
\end{aligned} \tag{S128}
$$

To obtain the E/I balance level for excitatory neurons in this network, we write the total excitatory input $I_i^{E,\mathrm{tot}}$ as the sum of different contributions,

$$
\begin{aligned}
\frac{I_i^{E,\mathrm{tot}}}{b} &= \sum_{k=1}^{P} (w_i^k \hat{x}^k + v_i^k \hat{y}^k) + \sum_{k=1}^{P} (w_i^k x^k + v_i^k y^k) && \text{(stimulus-specific, local)} \\
&+ 2 \left(\bar{\xi} \sum_{k=1}^{P} \hat{x}^k + \bar{\eta} \sum_{k=1}^{P} \hat{y}^k \right) && \text{(stimulus-specific, global)} \\
&+ \frac{2}{N} \left(\bar{\xi} \sum_{k=1}^{P} w_i^k + \bar{\eta} \sum_{k=1}^{P} v_i^k \right) \sum_{i=1}^{N} \phi(r_i^E) && \text{(stimulus-nonspecific, local)} \\
&+ 2\alpha \left(\bar{\xi}^2 + \bar{\eta}^2 \right) \sum_{i=1}^{N} \phi(r_i^E) + \bar{\xi} \sum_{k=1}^{P} x^k + \bar{\eta} \sum_{k=1}^{P} y^k. && \text{(stimulus-nonspecific, global)}
\end{aligned} \tag{S129}
$$

Taking the ratio between the stimulus-specific, local component and the net input to each excitatory neuron, we get,

$$
B_i^{E/I} = \left| \frac{I_i^R + I_i^F}{\delta I_i} \right| = \left| -1 + 2B_i \right|, \tag{S130}
$$

where I_i^F, I_i^R, δI_i and B_i are those defined in the original network model [without separation of E and I; Eq. (S77)]. Therefore, for moderate values of $B_i > 1/2$, up to a scaling factor and shift, the stimulus-specific, local component of the E/I balance level is the same as the balance level we analyzed in Figs. 2, 3. Note that in the range of α values analyzed in Fig. 2, the fraction of neurons with $B_i < 1/2$ is negligible in both match and mismatch conditions.

5.2. Interpolation via nonnegative matrix factorization

Solving for $\tilde{J}^{EI}$ and J^{IE} in Eq. (S128) is equivalent to a nonnegative matrix factorization problem [13]. Using the shifted, nonnegative weight vectors, we define the matrices Ξ, H, S,

$$\Xi = \frac{1}{N}\begin{pmatrix} \boldsymbol{\xi}^{1\top} \\ \vdots \\ \boldsymbol{\xi}^{P\top} \end{pmatrix} = \frac{1}{N}\begin{pmatrix} \xi_1^1 & \dots & \xi_N^1 \\ \vdots & \ddots & \vdots \\ \xi_1^P & \dots & \xi_N^P \end{pmatrix} \in \mathbb{R}^{P\times N},$$

$$H = \frac{1}{N}\begin{pmatrix} \boldsymbol{\eta}^{1\top} \\ \vdots \\ \boldsymbol{\eta}^{P\top} \end{pmatrix} = \frac{1}{N}\begin{pmatrix} \eta_1^1 & \dots & \eta_N^1 \\ \vdots & \ddots & \vdots \\ \eta_1^P & \dots & \eta_N^P \end{pmatrix} \in \mathbb{R}^{P\times N}, \qquad S = \begin{pmatrix} \Xi \\ H \\ \mathbf{0} \end{pmatrix} \in \mathbb{R}^{N\times N}. \tag{S131}$$

Throughout this section, we will assume $2P \leq N$, and '$\mathbf{0}$' pads with 0's such that S is a square matrix. Thus, the connectivity equation [Eq. (S128)] can be rewritten as,

$$\tilde{J}^{EI} J^{IE} = 2b(\Xi^\top \Xi + H^\top H) = b(\gamma+1)S^\top S. \tag{S132}$$

For each choice of a nonnegative matrix J^{IE}, the above equation has a nonnegative solution J^{EI} if and only if the convex cone formed by the row vectors of J^{IE} contains the convex cone formed by the row vectors of S [formally denoted as $\mathrm{cone}(J^{IE}) \supseteq \mathrm{cone}(S)$]. This condition can be derived from the definition of matrix multiplication [13]. Based on this condition, we identify a family of solutions $\{J^{EI}(\lambda),\ J^{IE}(\lambda)\}$ parameterized by $\lambda \in [0,1]$ as follows. At one end, we choose J^{IE} equal to the identity ($J^{IE}(\lambda = 0) = I_N$). At the other end, $J^{IE}(\lambda = 1) = S'$, where S' is defined such that its first $2P$ rows are the same as the nonzero rows of S and the rest of its rows are randomly sampled from the vectors $\boldsymbol{\xi}^k/N$, $\boldsymbol{\eta}^k/N$. This ensures that $\mathrm{cone}(S') \supseteq \mathrm{cone}(S)$. This family of solutions assumes that the number of inhibitory neurons equal to the number of excitatory neurons.

The firing-rates of inhibitory neurons are given by,

$$r_i^I(\lambda) \equiv \phi_I(h_i^I) = h_i^I = \sum_{j=1}^{N} J_{ij}^{IE}(\lambda) r_j^E. \tag{S133}$$

At the two ends, this reduces to,

$$\begin{aligned} r_i^I(\lambda = 0) &= r_i^E(0), \\ r_i^I(\lambda = 1) &= \begin{cases} \hat{x}^k + \frac{\bar{\xi}}{N}\sum_{i=1}^N \phi(h_i^E), & \text{if the } i\text{th row of } S' \text{ is } \boldsymbol{\xi}^{k\top} \\ \hat{y}^k + \frac{\bar{\eta}}{N}\sum_{i=1}^N \phi(h_i^E), & \text{if the } i\text{th row of } S' \text{ is } \boldsymbol{\eta}^{k\top} \end{cases} \end{aligned} \tag{S134}$$

Based on these equations, we call $\lambda = 0$ the 'private' solution and $\lambda = 1$ the 'internal

prediction' scenario. For $\lambda = 1$, the second term in $r_i^I(1)$ can be canceled by a global disinhibtory input. For intermediate λ's, it may seem natural to choose a linear interpolation between the two solutions, $J^{IE}(\lambda) = \lambda J^{IE}(1) + (1-\lambda)J^{IE}(0)$. We find however that this choice does not ensure that the solution for J^{EI} is nonnegative.

Instead, we choose E-to-I connectivity as follows. Two intermediate points within the segment $[0,1]$ are denoted as $\lambda = 0^+$ and $\lambda = 1^-$, thereby dividing the segment into three. At those points we choose J^{IE} to be,

$$J^{IE}(0^+) = \left(\begin{array}{c|c} \Xi_{P,2P} & \mathbf{0} \\ H_{P,2P} & \mathbf{0} \\ \hline \mathbf{0} & I_{N-2P} \end{array}\right), \qquad J^{IE}(1^-) = \left(\begin{array}{c|c} \Xi_{P,2P} & \Xi_{P,N-2P} \\ H_{P,2P} & H_{P,N-2P} \\ \hline \mathbf{0} & \mathrm{diag}(\boldsymbol{a}) \end{array}\right). \tag{S135}$$

Here, $\Xi_{P,2P}, H_{P,2P}$ consist of the first P rows and first $2P$ columns of Ξ and H, respectively; $\Xi_{P,N-2P}, H_{P,N-2P}$ consist of the first P rows and last $N-2P$ columns of Ξ and H, respectively; $\mathrm{diag}(\boldsymbol{a})$ is a diagonal matrix, with diagonal elements given by the $N-2P$ components of the vector $\boldsymbol{a}$ which is specified below. Again the $\mathbf{0}$'s are used for padding.

The interpolation of $J^{IE}(\lambda)$ from $\lambda = 0$ to $\lambda = 1$ thus consists of three regions:

(I) λ from 0 to 0^+: The upper left block of J^{IE} changes from an identity matrix to a matrix of stimulus input vectors.

(II) λ from 0^+ to 1^-: The upper and lower right blocks linearly interpolate the matrices shown in Eq. (S135). Results in the main text are taken from here.

(III) λ from 1^- to 1: The lower part of the matrix changes to contain stimulus vectors.

We start with solutions in Region (II) which we found to be the most relevant to the empirical measurements in [1], since we estimated $\lambda \approx 0.6$. Network properties for a range of λ values between 0 and 1 (Figs. 6, S8, S9, S10) are also based on the results in Region (II). The connectivity matrices $J^{EI}(\lambda)$ and $J^{IE}(\lambda)$ in Region (II) are given by,

$$\begin{aligned} J^{IE}(\lambda) &= \left(\begin{array}{c|c} \Xi_{P,2P} & \lambda\Xi_{P,N-2P} \\ H_{P,2P} & \lambda H_{P,N-2P} \\ \hline \mathbf{0} & (1-\lambda)I_{N-2P} + \lambda\mathrm{diag}(\boldsymbol{a}) \end{array}\right), \\ \tilde{J}^{EI}(\lambda) &= 2bN \left(\begin{array}{ccc} \Xi & H & \mathcal{J}(\lambda) \end{array} \right). \end{aligned} \tag{S136}$$

Here $\mathcal{J}(\lambda)$ is a $N \times (N-2P)$ matrix whose elements are given by

$$[\mathcal{J}(\lambda)]_{ij} = \frac{(1-\lambda)(\xi_i\xi_j + \eta_i\eta_j)}{(\lambda a_j + 1 - \lambda)N^2}, \quad i = 1,\ldots,N, \quad j = N-2P+1,\ldots,N. \tag{S137}$$

One can check that $\mathrm{cone}(J^{IE}(\lambda)) \supseteq \mathrm{cone}(S)$, and thus Eq. (S132) is satisfied and the elements of J^{EI} are nonnegative for every λ.

The interpolation in Region (I) requires smoothly 'morphing' the upper left block of the connectivity matrix involving Ξ and H to the identity matrix. This can be done by replacing the last row and last column with 0 and then setting the last diagonal element to be 1. Repeating this replacement P times yields the identity matrix. We note that in the low-dimensional case $[P = O(1)]$, this procedure only changes the E connections to P out of N inhibitory neurons. Thus its effect on the overall statistics of inhibitory neurons' activity is negligible. In the high-dimensional case $[P = O(N)]$, the distributions of neural activity and synaptic weights themselves change smoothly along this interpolation path. Similarly, in Region (III), we replace every row in the lower part of the matrix with one of the randomly sampled vectors that appear in the matrix S'.

5.3. Plasticity of inhibitory weights during learning

The interpolation solutions presented in the last section are valid for any set of positive real numbers a_i, $i = 2P + 1, \ldots, N$. In Fig. 6 we choose the a_i's as follows,

$$a_i(\mu) = \begin{cases} 1.4 + 12\mathrm{exp}[1.5 s_i(\mu)] & \text{if } s_i(\mu) \leq 0 \\ 0.002 & \text{if } 0 < s_i(\mu) < 0.97 \\ 2.002 & \text{if } s_i(\mu) \geq 0.97 \end{cases} \tag{S138}$$

where

$$s_i(\mu) = [r^E_{x,i}(\mu) - \langle r^E_x(\mu)\rangle][r^E_{xy,i}(\mu) - \langle r^E_{xy}(\mu)\rangle]. \tag{S139}$$

Here $r^E_{x,i}(\mu)$ and $r^E_{xy,i}(\mu)$ are the firing-rates of the i-th excitatory neuron in the x-only mismatch and match conditions for a given value of μ. $\langle r^E_x(\mu)\rangle$ and $\langle r^E_{xy}(\mu)\rangle$ are the average firing-rates over all the E neurons in the two conditions. This mathematical form for a_i is chosen to match the experimental data on fast spiking neurons (Fig. 6c,d).

To track individual synapses during learning, we generate the kth stimulus input vectors $\boldsymbol{\xi}^k$ and $\boldsymbol{\eta}^k$ as follows: (1) We first generate two independent isotropic Gaussian vectors $\boldsymbol{a}^k_0$, $\boldsymbol{b}^k_0$, with mean equal to 3 and standard deviation equal to 1; (2) Then we form the a linear combination to generate two correlated Gaussian random variables,

$$\boldsymbol{a}^k = \boldsymbol{a}^k_0, \qquad \boldsymbol{b}^k = \mu \boldsymbol{a}^k_0 + \sqrt{1-\mu^2}\boldsymbol{b}^k_0. \tag{S140}$$

(3) Finally, we clip both variables to positive and define them as $\boldsymbol{\xi}^k$ and $\boldsymbol{\eta}^k$. In this case, the resulting vectors $\boldsymbol{w}^k$ and $\boldsymbol{v}^k$ [Eq. (S126)] will be approximately correlated Gaussian variables with mean 0. These procedures are used to produce the plots in Fig. 6c,e,f,g.

6. PARAMETER VALUES USED IN THE FIGURES

Unless specified, in all the main and supplementary figures, $\boldsymbol{w}^k$ and $\boldsymbol{v}^k$ have joint Gaussian distribution and satisfy Eq. (S9). The number of neurons in the network is $N = 2000$.

Figure 1: We set $\alpha = 0$, $\theta = 0$ and $b = 150$ throughout this figure.

Panel b: We use Eq. (S72) to generate $N = 2000$ samples of 2D random variables (I_x, I_{xy}) and compute the corresponding firing-rates.

Panel d: The theory lines for the Pearson correlation between different stimulus conditions are calculated from Eqs. (S71, S73, S76). The simulation points are calculated by sampling the neurons' firing-rates as described in the Panel b caption. As each vector represents the mean-subtracted firing-rate vectors, the cosine of the angle is equivalent to the Pearson correlation coefficient between the original firing-rate vectors.

Panel f: The firing-rate distribution on the left ('Our model') is generated in the same way as in Panel b. As the neural responses to two stimulus-pairs are mutually independent at $\alpha = 0$, the joint distribution is a product of the corresponding marginal distributions. The firing-rate distribution on the right ('Segregated model') is generated by using the same marginal distributions (as in the plot of 'Our model'), but adding a nonzero correlation (which equals to 0.9) in the input variables (I_x, I_{xy}) that are used to calculate the firing-rates.

Figure 2: We set $\theta = 0$ throughout this figure.

Panel b: $\alpha = 0$, $b = 150$. The 'Early' and 'Late' plots for balance level distribution are calculated at $\mu = 0$ and $\mu = 0.9$ respectively.

Panel d: $\alpha = 0$, $b = 150$. For SVM classification, stimulus inputs in the mismatch condition are generated from Gaussian mixtures centered at $(0, 1)$ and $(1, 0)$, both of which are isotropic and have variance 0.05. Similar Gaussian mixtures are used for stimulus input in the match condition, except that the centers are at $(0, 0)$ and $(1, 1)$. The SVM model is fitted using the Matlab function 'fitcsvm'. The classification error is calculated via the matlab functions 'crossval' and 'kfoldLoss'.

Panel e: This figure panel is an illustration and the parameters are $\alpha = 0$, $\mu = 0.7$.

Panel f: The threshold on the firing-rate for determining the optimal b is chosen such that at $\alpha = 0$, the optimal balance level is the same as the one fitted to experimental data [14] in Fig. 3 ($B^\star \approx 162$).

Figure 3: We fit both sets of experimental data [1, 14] using Eq. (8) (Methods).

Figure 4:

Panel b: $\alpha = 0$, $b = 150$.

Panel c: The values of b in both plots are chosen to be at the optimal values.

Panel d: Left: fraction of mixed-representation neurons among all PE neurons for the stimulus pair 1. Right: fraction of purely prediction error neurons among all neurons that are classified as PE neuron for at least 1 stimulus-pair. Error bars indicate standard deviations over 200 instances of the network. Dedicated PE neurons are defined as those neurons that are classified as having PE responses for at least one stimulus-pair while not being classified as having R responses for any of the other stimulus-pairs. The threshold for defining response types was based on neural activity statistics at $\alpha = 0$ and was used for all values of α.

Panel f: We set $b = 189$, which is the value extracted from the data [1]. The sparsity levels are defined as the fraction of active neurons in the network and changed by varying the firing-rate threshold θ in the network model. The threshold values corresponding to the three plotted curves are $\theta = 4.5, 6.5, 21.5$.

Figure 5: $\theta = 0$ throughout this figure. The number of neurons in the network is 600. All the error bars are computed based on 10 random samples of synaptic weight vectors. The dynamics of the network is obtained by simulating the ODEs [Eq. (S29)] for total time $t = 1$. Panel b, c: $\alpha = 0$, $b = 150$. Panel d: The threshold for classifying function cell-types is the same as in Fig. 4. The early and late time points are $t = 0.02$ and $t = 0.3$. The b values used for different stimulus dimensions are the same as the optimal b values obtained as in the steady state response (Fig. 2).

Figure 6: We set $\theta = 0$, $J^{II} = 0$ throughout this figure. Before and after learning correspond to $\mu = 0$ and $\mu = 0.97$. During learning, the functional cell types of a specific E or I neuron in the network might change. The cell-type-specific synaptic weight statistics shown in Fig. 5f,g only include synapses whose pre- and postsynaptic neurons maintain their identity throughout learning. Other parameter values can be found in §5.3.

Figure 7: $\theta = 0$ throughout this figure. The number of neurons for each module is 400. All the error bars are computed based on 30 random samples of synaptic weight vectors. The steady state of the network is obtained by simulating the ODEs [Eq. (S29)] for total time $t = 4$.

Panel b : $\alpha = 0$, $\mu = 0.97$. The colormap indicates the firing rate averaged over all neurons in all modules in the x-only mismatch condition.

Panel c: $b_1 = b_3 = 50$ and $b_2 = 190$ are the values at the star position in panel b. $\mu = 0.97$.

Panel d-h: $b_1 = b_3 = 50$ and $b_2 = 190$ are the values at the star position in panel b.

Figure S1:

Panel a: $\alpha = 0$, $b = 150$.

Panel c: The threshold on firing-rate for determining optimal b is chosen such that at $\alpha = 0$, the optimal balance level is the same as the one fitted to experimental data [14] in Fig. 3 ($B^\star \approx 162$). This threshold remains fixed for different values of α.

Figure S3: Throughout this figure, $\alpha = 0$, $b = 150$.

Figure S4: Throughout this figure, $\mu = 0.97$, $b = 150$.

Figure S5: The threshold value corresponding to the model curve is $\theta = -20$. We set $b = 189$, which is the value extracted from the data [1].

Figure S6: $\theta = 0$ throughout this figure. The number of neurons in the network is 600. All the error bars are computed based on 10 random samples of synaptic weight vectors. The dynamics of the network is obtained by simulating the ODEs [Eq. (S29)] for total time $t = 6$. Panel a, c: $\alpha = 0$, $b = 150$. Panel b: $\alpha = 0$, $b = 150$. The Δ similarity (peak) is computed as the difference between the peak values of the cosine similarity with respect to $\boldsymbol{r}_{xy}$ during the interval of y input and the interval of x input. Panel d, right: $\alpha = 0$, $\mu = 0.97$. The are is computed as the area below the balance level curve but above the steady state value for y-only condition. Panel e, f: The threshold for classifying function cell-types is the same as in Fig. 4. The b values used for different stimulus dimensions are the same as the optimal b values obtained as in the steady state response (Fig. 2). The total number of neurons are from 50 repeats of the random networks.

Figure S7: $\theta = 0$ throughout this figure. The number of neurons in the network is 600. All the error bars are computed based on 10 random samples of synaptic weight vectors. The dynamics of the network is obtained by simulating the ODEs [Eq. (S29)] for pruned synaptic connectivity. Panel b, e, g: $\alpha = 0$, $b = 150$, $f_w = 0.7$. Panel c: $\alpha = 0$, $b = 150$. The power spectrum of voltage level is computed over time window of length 100. Panel d: $\alpha = 0$, $b = 150$ Panel f, h: The threshold for classifying function cell-types is the same as in Fig. 4. The b values used for different stimulus dimensions are the same as the optimal b values obtained as in the steady state response (Fig. 2).

Figure S8: Throughout this figure, we set $\alpha = 0$, $b = 150$.

Figure S9 and S10: We set $\theta = 0$, $J^{II} = 0$ throughout these figures. Before and after learning correspond to $\mu = 0$ and $\mu = 0.97$. Other parameter values are the same as in Fig. 6.

Figure S11: $\theta = 0$ throughout this figure. The number of neurons for each module is 400. All the error bars are computed based on 50 random samples of synaptic weight vectors. The steady state of the network is obtained by simulating the ODEs for a

total time $t = 6$. $b_1 = b_3 = 50$ and $b_2 = 190$.

Panel c: $\mu = 0$ is used for before learning and $\mu = 0.97$ is for after learning. The SVM model for decoding is fitted using the Matlab function `fitcsvm`. The classification error is calculated via the Matlab functions `crossval` and `kfoldLoss`.

---

[1] N. J. Audette, W. Zhou, A. La Chioma, and D. M. Schneider, "Precise movement-based predictions in the mouse auditory cortex," *Current Biology*, vol. 32, no. 22, pp. 4925–4940, 2022.

[2] M. V. Srinivasan, S. B. Laughlin, and A. Dubs, "Predictive coding: a fresh view of inhibition in the retina," *Proceedings of the Royal Society of London. Series B. Biological Sciences*, vol. 216, no. 1205, pp. 427–459, 1982.

[3] R. P. Rao and D. H. Ballard, "Predictive coding in the visual cortex: a functional interpretation of some extra-classical receptive-field effects," *Nature Neuroscience*, vol. 2, no. 1, pp. 79–87, 1999.

[4] A. M. Bastos, W. M. Usrey, R. A. Adams, G. R. Mangun, P. Fries, and K. J. Friston, "Canonical microcircuits for predictive coding," *Neuron*, vol. 76, no. 4, pp. 695–711, 2012.

[5] K. Friston and S. Kiebel, "Predictive coding under the free-energy principle," *Philosophical transactions of the Royal Society B: Biological sciences*, vol. 364, no. 1521, pp. 1211–1221, 2009.

[6] L. P. Jiang and R. P. Rao, "Predictive coding theories of cortical function," in *Oxford Research Encyclopedia of Neuroscience*, Oxford Univ. Press, 2022.

[7] F. A. Mikulasch, L. Rudelt, and V. Priesemann, "Local dendritic balance enables learning of efficient representations in networks of spiking neurons," *Proceedings of the National Academy of Sciences*, vol. 118, no. 50, p. e2021925118, 2021.

[8] F. A. Mikulasch, L. Rudelt, M. Wibral, and V. Priesemann, "Where is the error? hierarchical predictive coding through dendritic error computation," *Trends in Neurosciences*, vol. 46, no. 1, pp. 45–59, 2023.

[9] M. Mézard, G. Parisi, and M. A. Virasoro, *Spin glass theory and beyond: An Introduction to the Replica Method and Its Applications*, vol. 9. World Scientific Publishing Company, 1987.

[10] R. Kuhn and S. Bos, "Statistical mechanics for neural networks with continuous-time dynamics," *Journal of Physics A: Mathematical and General*, vol. 26, no. 4, p. 831, 1993.

[11] N. J. Audette and D. M. Schneider, "Stimulus-specific prediction error neurons in mouse auditory cortex," *Journal of Neuroscience*, vol. 43, no. 43, pp. 7119–7129, 2023.

[12] T. Haga and T. Fukai, "Extended temporal association memory by modulations of inhibitory circuits," *Physical Review Letters*, vol. 123, no. 7, p. 078101, 2019.

[13] N. Gillis, *Nonnegative Matrix Factorization.* SIAM, 2020.

[14] R. Jordan and G. B. Keller, "Opposing influence of top-down and bottom-up input on excitatory layer 2/3 neurons in mouse primary visual cortex," *Neuron*, vol. 108, no. 6, pp. 1194–1206, 2020.
